# Impact of birth weight on cardiovascular disease and mediating role of metabolic traits: a Mendelian randomisation study

**DOI:** 10.1136/openhrt-2025-003561

**Published:** 2025-10-31

**Authors:** Juncheng Zhuang, Shuhao Chen, Jinping Long, Ding Ding, Lawrence T Lam, Jie Li, Ran An

**Affiliations:** 1 Faculty of Medicine, Macau University of Science and Technology, Taipa, Macao SAR, China; 2 Global Health Research Center, Guangdong Provincial People’s Hospital, Guangdong Academy of Medical Sciences, Southern Medical University, Guangzhou, China; 3 Department of Epidemiology, Southern Medical University, Guangzhou, China; 4 Research Management Department, Guangdong Provincial People’s Hospital, Guangdong Academy of Medical Sciences, Southern Medical University, Guangzhou, China; 5 Faculty of Medicine and Health, The University of Sydney, Sydney, New South Wales, Australia; 6 Faculty of Health, University of Technology Sydney, Sydney, New South Wales, Australia; 7 Medical Research Center Division of Laboratory Medicine, Guangdong Provincial People's Hospital-Guangdong Academy of Medical Sciences, Guangzhou, China

**Keywords:** RISK FACTORS, Metabolic Syndrome, Coronary Artery Disease, Epidemiology

## Abstract

**Background:**

Birth weight (BW) has been linked to cardiometabolic diseases, but causal associations with a comprehensive range of cardiovascular outcomes and underlying metabolic mechanisms remain unclear.

**Methods:**

We applied a two-sample Mendelian randomisation (MR) approach to evaluate causal relationships between genetically predicted BW and 16 distinct cardiovascular diseases (CVD). We further conducted a two-step MR mediation analysis to quantify the mediating roles of 24 metabolic traits covering body composition, glucose metabolism, lipid metabolism, blood pressure, fatty acids and amino acids.

**Results:**

Genetically lower BW was associated with higher risks of coronary heart disease (OR 0.72, 95% CI 0.65 to 0.81), myocardial infarction (OR 0.71, 95% CI 0.63 to 0.80) and angina pectoris (OR 0.81, 95% CI 0.72 to 0.90). These effects were partly mediated by type 2 diabetes, systolic blood pressure, total cholesterol and triglycerides, explaining 11.76–33.33% of the total associations. In contrast, genetically higher BW increased the risk of aortic aneurysm (OR 1.46, 95% CI 1.21 to 1.75), venous thromboembolism (OR 1.22, 95% CI 1.09 to 1.36) and atrial fibrillation (OR 1.34, 95% CI 1.21 to 1.48). These associations were partly explained by body composition traits, with appendicular lean mass and body mass index mediating 10.53–26.32% of the effect on aortic aneurysm, 15.79–68.42% of the effect on venous thromboembolism and 10.34–58.62% of the effect on atrial fibrillation.

**Conclusions:**

Our study provides robust evidence of distinct causal pathways linking BW with adult cardiovascular risks through specific metabolic mediators. These findings highlight the importance of optimal fetal growth and lifelong metabolic health management as critical strategies to reduce CVD burden.

**WHAT IS ALREADY KNOWN ON THIS TOPIC**

- Birth weight (BW) is considered a key indicator of intrauterine growth and has been associated with cardiovascular disease (CVD) risk in numerous observational studies.
- Previous Mendelian randomisation studies have provided preliminary evidence of causality between BW and certain CVDs; they have largely been limited in scope and have not systematically assessed the mediating roles of metabolic traits across a broad spectrum of cardiovascular outcomes.

**WHAT THIS STUDY ADDS**

- Both low BW and high BW have distinct impacts on adult cardiovascular risk through specific metabolic pathways. Low BW increases the risk of coronary events via diabetes, hypertension and dyslipidaemia, while high BW raises the risk of aortic aneurysm, venous thromboembolism and atrial fibrillation through increased body size.

**HOW THIS STUDY MIGHT AFFECT RESEARCH, PRACTICE OR POLICY**

- BW can serve as a simple, early-life marker to identify individuals at higher lifetime risk for specific CVDs, enabling timely and tailored preventive strategies.
- Managing key metabolic traits—such as blood pressure, lipids, glucose levels and body composition—based on an individual’s BW profile may help mitigate cardiovascular risk more effectively across the lifespan.

## Introduction

 Cardiovascular diseases (CVD) remain the leading cause of morbidity and mortality worldwide, with a substantial proportion of risk attributable to metabolic disturbances and early-life exposures.^1^ The Developmental Origins of Health and Disease (DOHaD) hypothesis posits that suboptimal intrauterine environments trigger maladaptive physiological and metabolic adaptations, thereby predisposing individuals to lifelong cardiometabolic disorders.^2^ Birth weight (BW), a well-validated marker of intrauterine growth and fetal development, has consistently been linked to adult cardiometabolic health.^3^,^7^ However, interpretation of observational studies is limited by residual confounding, measurement error and potential reverse causality.

Mendelian randomisation (MR), which leverages genetic variants as instrumental variables, provides an approach to strengthen causal inference. Prior MR studies have contributed preliminary evidence for a causal relationship between BW and selected cardiovascular outcomes—for example, lower BW with myocardial infarction and higher BW with atrial fibrillation.^8 9^ Yet, these investigations were restricted to a small number of endpoints, leaving it unclear how the significant heterogeneity across the spectrum of CVDs influences the BW–CVD association. Different CVD subtypes may involve distinct pathophysiological mechanisms, metabolic pathways, genetic architectures and environmental determinants. Without a systematic assessment, the overall landscape of how BW shapes cardiovascular health remains incompletely understood.

In addition, while metabolic traits are widely thought to mediate the BW–CVD relationship, their precise contributions have not been quantified across multiple outcomes.^10 11^ Identifying and quantifying such mediators is crucial, as it can elucidate the biological mechanism linking fetal growth to adult cardiovascular risk and inform precision prevention strategies.

To address these gaps, we conducted a comprehensive two-step MR study to evaluate the causal effects of BW on 16 distinct CVD outcomes, encompassing both atherosclerotic and non-atherosclerotic diseases. Furthermore, we applied a two-step MR mediation framework to quantify the roles of 24 metabolic traits spanning body composition, glucose metabolism, lipid metabolism, blood pressure, amino acids and fatty acids. By systematically mapping the distinct causal pathways through which BW influences cardiovascular risk, our study provides novel insights into the complex development origins of CVD and highlights key metabolic mediators that may inform future prevention and intervention strategies.

## Methods

### Study design

**Figure 1:**
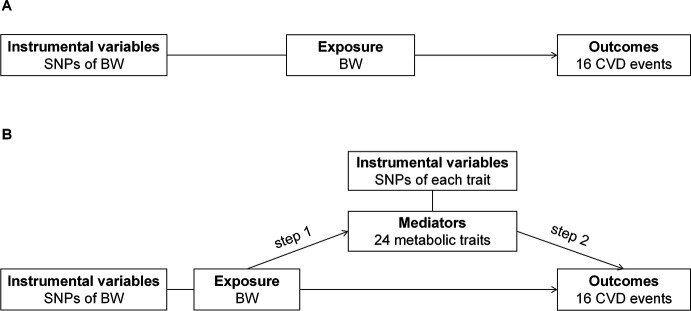
Overview of the study design. (**A**) A two-sample Mendelian randomisation (MR) analysis was conducted to assess the causal effect of birth weight (BW) on 16 cardiovascular disease (CVD) outcomes. (**B**) A two-step MR approach was used for mediation analysis. In step 1, the causal effect of BW on potential mediators was evaluated. In step 2, the causal effect of these mediators on CVD outcomes was estimated. Solid lines represent single nucleotide polymorphisms (SNPs) used as instrumental variables, while solid arrows indicate hypothesized causal pathways to be tested.

An overview of the study design is presented in figure 1. Initially, we employed two-sample MR analyses to investigate the causal relationship between BW and 16 distinct CVD outcomes. Subsequently, we used a two-step MR approach to identify and quantify the mediating roles of 24 potential metabolic traits in the pathway from BW to specific CVD outcomes. In step 1, the causal effects of BW on candidate mediators were assessed using two-sample MR analyses. In step 2, the causal effects of each mediator on CVD outcomes, independent of BW, were estimated using multivariable Mendelian randomisation (MVMR) analyses. The reporting of this study adheres to the Strengthening the Reporting of Observational Studies in Epidemiology using Mendelian Randomization (STROBE-MR) guidelines. MR analyses were based on three core assumptions: (1) instrumental variants are robustly associated with the exposure; (2) instrumental variants are independent of confounders affecting the exposure–outcome association; and (3) instrumental variants affect outcomes exclusively through the exposure.^12^

### Patient and public involvement

The study was conducted without engagement of patients or the public in its development or operational stages.

### Data sources and genetic instruments

The data sources used for exposure, mediators and outcomes are detailed in table 1. Genome-wide association study (GWAS) summary statistics for BW, metabolic traits and CVD outcomes were obtained from publicly available databases, including regression coefficients, SEs, effect alleles, reference alleles and allele frequencies.

**Table 1:** Summary of GWAS datasets used in the Mendelian randomisation analyses

Phenotype	Sample size (overall or case/control)	Ancestry	Unit	PMID/GWAS ID	Consortium
**Exposure**					
Birth weight	298 142	European	1 SD	31043758	EGG
**Mediator**					
Body composition					
Body mass index	681 275	European	1 SD	30124842	GIANT
Height	4 080 687	European	1 SD	36224396	GIANT
Appendicular lean mass	450 243	European	1 SD	33097823	UK Biobank
Glucose metabolism					
Type 2 diabetes	80 154/853 816	European	Log OR	35551307	DIAGRAM
Fasting glucose	281 416	European	mmol/L	34059833	MAGIC
2-hour glucose	85 916	European	mmol/L	34059833	MAGIC
Lipid and lipoprotein					
Total cholesterol	1 320 016	European	mg/dL	37237109	GLGC
HDL cholesterol	1 320 016	European	mg/dL	37237109	GLGC
LDL cholesterol	1 320 016	European	mg/dL	37237109	GLGC
Triglycerides	1 320 016	European	mg/dL	37237109	GLGC
Blood pressure					
Hypertension	137 312/316 345	European	Log OR	36653562	FinnGen
Systolic blood pressure	757 601	European	mm Hg	30224653	ICBP
Diastolic blood pressure	757 601	European	mm Hg	30224653	ICBP
Fatty acid					
Omega-3 fatty acids	114 999	European	1 SD	35692035	UK Biobank
Omega-6 fatty acids	114 999	European	1 SD	35692035	UK Biobank
Docosahexaenoic acid	114 999	European	1 SD	35692035	UK Biobank
Linoleic acid	114 999	European	1 SD	35692035	UK Biobank
Amino acid					
Isoleucine	115 075	European	1 SD	met-d-Ile	UK Biobank
Leucine	115 074	European	1 SD	met-d-Leu	UK Biobank
Valine	115 048	European	1 SD	met-d-Val	UK Biobank
Phenylalanine	115 025	European	1 SD	met-d-Phe	UK Biobank
Tyrosine	114 911	European	1 SD	met-d-Tyr	UK Biobank
Alanine	115 074	European	1 SD	met-d-Ala	UK Biobank
Glycine	114 972	European	1 SD	met-d-Gly	UK Biobank
**Outcome**					
Atherosclerotic diseases					
Coronary heart disease	60 801/123 504	Mixed	Log OR	26343387	CARDIoGRAMplusC4D
Myocardial infarction	43 676/128 199	Mixed	Log OR	26343387	CARDIoGRAMplusC4D
Angina pectoris	40 366/378 019	European	Log OR	36653562	FinnGen
Ischaemic stroke	40 585/406 111	European	Log OR	29531354	MEGASTROKE
Peripheral artery disease	11 924/288 638	European	Log OR	36653562	FinnGen
Infection-related diseases					
Rheumatic heart disease	1018/452 616	European	Log OR	36653562	FinnGen
Myocarditis	1847/231 952	European	Log OR	36653562	FinnGen
Pericarditis	1203/342 690	European	Log OR	36653562	FinnGen
Endocarditis	1140/342 690	European	Log OR	36653562	FinnGen
Other cardiovascular diseases					
Aortic aneurysm	8125/381 977	European	Log OR	36653562	FinnGen
Heart failure	33 250/394 758	European	Log OR	36653562	FinnGen
Intracerebral haemorrhage	4542/408 782	European	Log OR	36653562	FinnGen
Subarachnoid haemorrhage	3814/408 928	European	Log OR	36653562	FinnGen
Venous thromboembolism	23 367/430 366	European	Log OR	36653562	FinnGen
Non-rheumatic valvular disease	25 200/342 690	European	Log OR	36653562	FinnGen
Atrial fibrillation	60 620/970 216	European	Log OR	36653562	FinnGen

CARDIoGRAMplusC4D, Coronary ARtery Disease Genome wide Replication and Meta-analysis plus The Coronary Artery Disease Genetics; DIAGRAM, Diabetes Genetic Replication and Meta-analysis; EGG, Early Growth Genetics; GIANT, Genetic Investigation of Anthropometric Traits; GLGC, Global Lipids Genetics Consortium; GWAS, genome-wide association study; HDL, high-density lipoprotein; ICBP, International Consortium for Blood Pressure; LDL, low-density lipoprotein; MAGIC, Meta-Analyses of Glucose and Insulin-related Traits Consortium; MEGASTROKE, Multi-ancestry Genome-wide Association Study of Stroke; PMID, PubMed ID.

### Birth weight

Instrumental variables for BW were identified from the GWAS meta-analysis conducted by Warrington *et al*, encompassing 80 745 individuals from the Early Growth Genetics consortium and 217 397 individuals from the UK Biobank.^13^ All BW measurements were standardised to Z-scores, adjusted for sex, gestational duration and ancestry-informative principal components. Genome-wide significant (p<5×10^−8^), independent (r²<0.001) single nucleotide polymorphisms (SNPs) were selected as instrumental variables. Instrument strength for BW was robust with F-statistics exceeding 10.

### Candidate mediators

24 metabolic traits were considered, grouped into six categories: body composition, glucose metabolism, lipid metabolism, blood pressure, fatty acids and amino acids. GWAS details, including sample sizes, ancestry, units, consortium sources and GWAS IDs, are presented in table 1.

Candidate mediators were selected based on three criteria: (1) significant causal association between BW and mediator, (2) significant causal association between mediator and specific CVD outcomes independent of BW and (3) directional consistency between the total effect of BW on CVD and the indirect effect through the mediator. For each mediator, genome-wide significant SNPs (p<5×10^−8^) were identified, ensuring linkage disequilibrium independence (r²<0.001 within 10 000 kb windows). In MVMR analyses, after identifying genome-wide significant SNPs for BW and each mediator, these SNPs were combined and pruned to retain only independent variants (r²<0.001 within 10 000 kb windows).

### Outcomes

The study investigated 16 specific CVD outcomes classified into three categories: atherosclerotic diseases (coronary heart disease, myocardial infarction, angina pectoris, ischaemic stroke, peripheral artery disease), infection-related diseases (rheumatic heart disease, myocarditis, pericarditis, endocarditis) and others (aortic aneurysm, heart failure, intracerebral haemorrhage, subarachnoid haemorrhage, venous thromboembolism, non-rheumatic valvular disease, atrial fibrillation). Genetic instruments for outcomes were sourced from large GWAS meta-analyses by the FinnGen, Coronary ARtery Disease Genome wide Replication and Meta-analysis plus The Coronary Artery Disease Genetics (CARDIoGRAMplusC4D) and Multi-ancestry Genome-wide Association Study of Stroke (MEGASTROKE) consortia.

### Statistical analyses

Two-sample MR analyses were initially conducted to assess causal associations between BW and CVD outcomes using Wald ratio estimates calculated for individual SNPs, followed by inverse-variance weighted (IVW) meta-analysis for pooled effect estimation.^14^

A two-step MR mediation analysis was then employed.^15^ In the first step, the IVW method quantified the causal effect (β₁) of BW on each mediator. In the second step, MVMR was used to estimate the causal effect (β₂) of each mediator on CVD outcomes, adjusted for BW.^16^ The indirect effect was computed as the product of β₁ and β₂, and the proportion mediated was derived by dividing the indirect effect by the total effect, with SEs calculated using the delta method.^17^

To ensure robustness, multiple sensitivity analyses were performed using weighted median, weighted mode, MR-Egger and MR-pleiotropy residual sum and outlier (MR-PRESSO) methods, which accommodate different assumptions regarding pleiotropy and instrumental validity. MR-Egger intercept analysis assessed horizontal pleiotropy,^18^ while Cochran’s Q test evaluated heterogeneity. Leave-one-out analyses assessed the influence of individual SNPs on the results. To avoid bias due to overlapping SNPs, we performed sensitivity analyses in which variants that were genome-wide significant for both BW and the respective mediators were excluded prior to mediation analyses.

Bonferroni-corrected thresholds were applied for statistical significance: p<0.05/16 for BW–CVD associations, p<0.05/24 for BW–mediator associations and p<0.05/96 for mediator–CVD associations. Results were presented as ORs and β coefficients with 95% CIs.

All analyses were conducted using R V.4.2.3, employing the ‘Two-SampleMR’ and ‘MendelianRandomization’ packages.

## Results

### Causal associations between BW and CVDs

**Figure 2:**
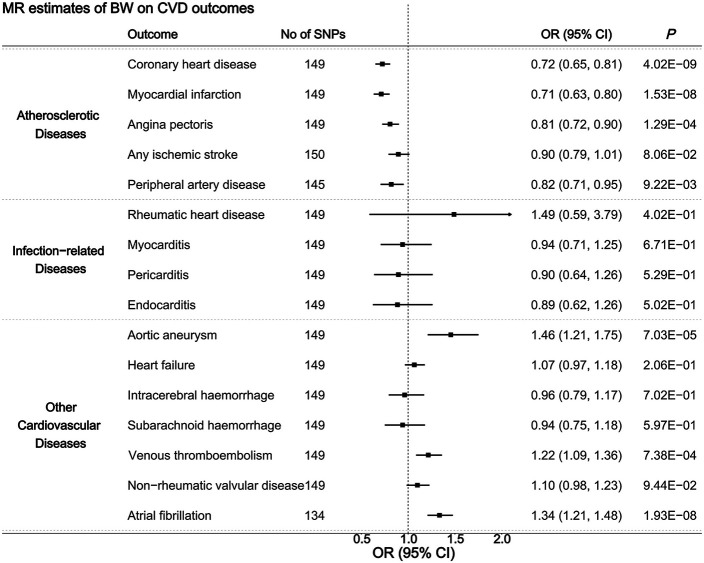
Causal associations between BW and 16 CVD outcomes. Causal estimates were derived using the IVW method. A Bonferroni-corrected p value threshold of <0.0031 was considered statistically significant. BW, birth weight; CVD, cardiovascular disease; IVW, inverse-variance weighted; MR, Mendelian randomisation; SNPs, single nucleotide polymorphisms.

**Figure 3:**
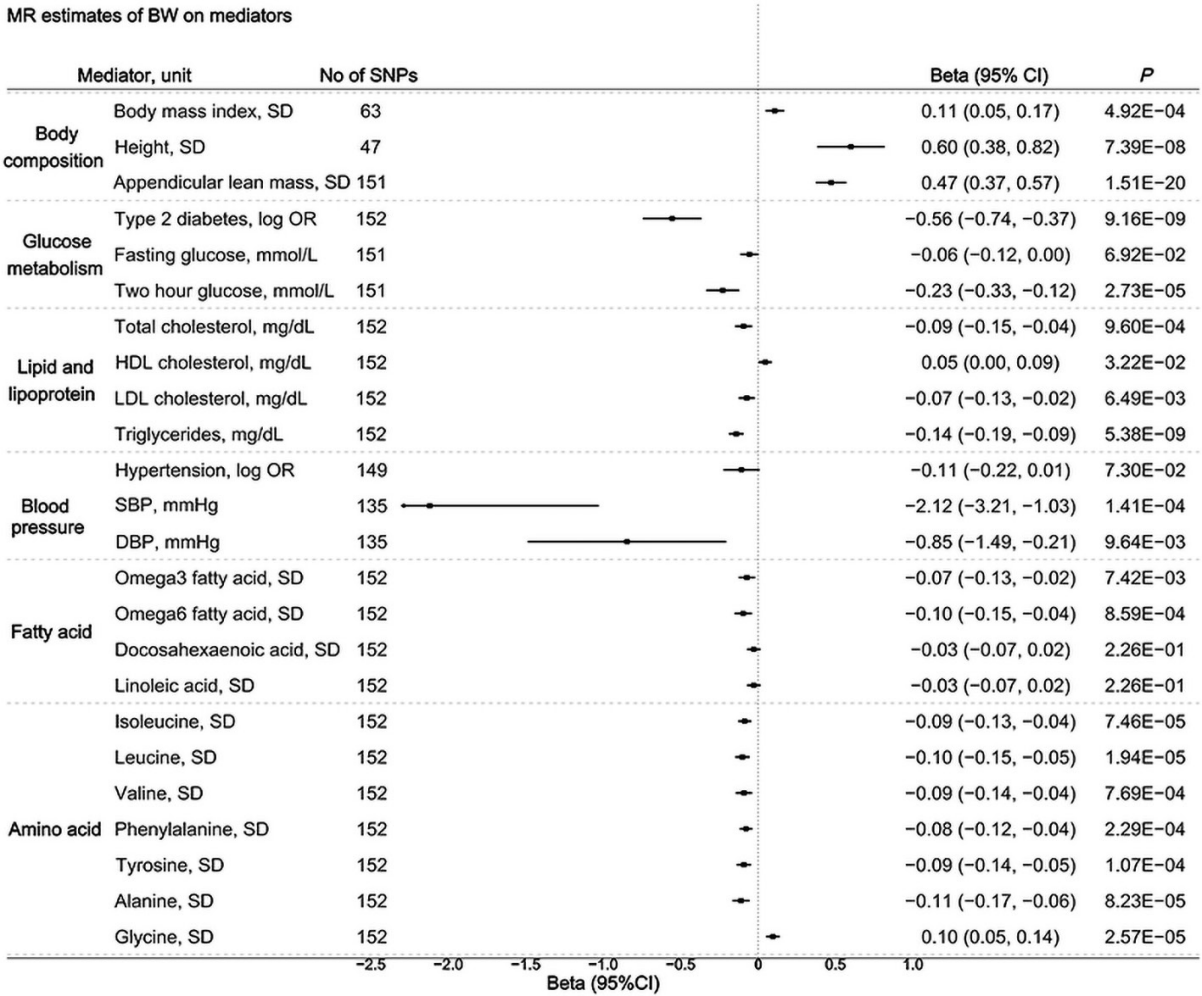
Causal associations between BW and 24 potential mediators. Causal estimates were obtained using the IVW method. A Bonferroni-corrected p value <0.0021 was considered statistically significant. BW, birth weight; DBP, diastolic blood pressure; HDL, high-density lipoprotein; IVW, inverse-variance weighted; LDL, low-density lipoprotein; MR, Mendelian randomisation; SBP, systolic blood pressure; SNPs, single nucleotide polymorphisms.

**Figure 4:**
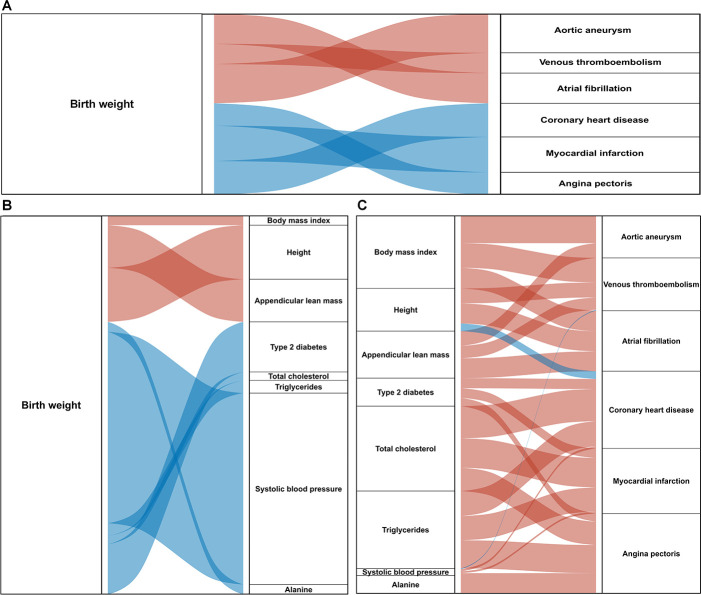
Sankey diagrams illustrating the causal pathways from birth weight (BW) to cardiovascular diseases (CVD) through potential mediators. (**A**) Sankey diagrams illustrating the causal association between BW and specific CVDs. (**B**) Sankey diagrams illustrating the causal association between BW and qualified mediators. (**C**) Sankey diagrams illustrating the causal association between qualified mediators and specific CVD. In all panels, red and blue flows indicate positive and negative associations, with widths corresponding to effect sizes.

Two-sample MR analyses revealed significant causal associations between BW and several CVD outcomes after applying Bonferroni correction (figure 2). Specifically, genetically predicted higher BW was associated with decreased risks of coronary heart disease (OR 0.72, 95% CI 0.65 to 0.81), myocardial infarction (OR 0.71, 95% CI 0.63 to 0.80) and angina pectoris (OR 0.81, 95% CI 0.72 to 0.90). Conversely, genetically predicted higher BW was positively associated with the risks of aortic aneurysm (OR 1.46, 95% CI 1.21 to 1.75), venous thromboembolism (OR 1.22, 95% CI 1.09 to 1.36) and atrial fibrillation (OR 1.34, 95% CI 1.21 to 1.48). Sensitivity analyses (weighted median, MR-PRESSO) confirmed robust results without evidence of pleiotropy, and no single SNP had a disproportionate effect (online supplemental table 1 and figures 1–16).

### Causal associations between BW and potential mediators

BW was significantly associated with 16 of the 24 tested metabolic mediators after Bonferroni correction. Specifically, higher BW was causally linked to greater body composition traits, including body mass index (BMI; β 0.11, 95% CI 0.05 to 0.17), height (β 0.60, 95% CI 0.38 to 0.82) and appendicular lean mass (β 0.47, 95% CI 0.37 to 0.57), as well as higher glycine levels (β 0.10, 95% CI 0.05 to 0.14). Additionally, higher BW was associated with reduced risks of type 2 diabetes (OR 0.57, 95% CI 0.48 to 0.69), lower systolic blood pressure (β −2.12, 95% CI −3.21 to −1.03) and improved lipid profiles, including lower total cholesterol (β −0.09, 95% CI −0.15 to −0.04) and triglycerides (β −0.14, 95% CI −0.19 to −0.09). Amino acids, including isoleucine, leucine, valine, phenylalanine, tyrosine and alanine, were also inversely associated with BW (figure 3, online supplemental table 2). Sensitivity analyses using MR-Egger, MR-PRESSO, Cochran’s Q tests and the exclusion of overlapping SNPs between BW and mediators confirmed the robustness of these results (online supplemental table 2).

### Causal associations between potential mediators and CVDs

Eight mediators were identified as causally associated with the specified CVD outcomes. BMI, type 2 diabetes, total cholesterol, triglycerides and systolic blood pressure were positively associated with coronary heart disease, myocardial infarction and angina pectoris, with ORs ranging from 1.03 to 1.65. Alanine was also positively associated with angina pectoris. In contrast, height exhibited an inverse relationship with coronary heart disease (OR 0.89, 95% CI 0.85 to 0.94). Moreover, height, BMI and appendicular lean mass showed significant positive associations with aortic aneurysm, venous thromboembolism and atrial fibrillation, with ORs ranging from 1.20 to 1.50. Triglycerides were positively associated with aortic aneurysm, while systolic blood pressure showed an inverse association with venous thromboembolism but a positive association with atrial fibrillation. Sensitivity analyses using the weighted median method, MR-Egger regression and exclusion of overlapping SNPs further supported the robustness of these associations (online supplemental table 3).

### Mediation analysis of metabolic traits between BW and CVDs

The mediation analysis identified and quantified the mediating roles of eight metabolic traits linking BW to six CVD outcomes. For aortic aneurysm, venous thromboembolism and atrial fibrillation, BMI and appendicular lean mass were significant mediators, accounting for 10.34–48.28% of the total effect. Height also played a major mediation role, accounting for 68.42% of the effect on venous thromboembolism, 58.62% on atrial fibrillation and 21.88% on coronary heart disease. In contrast, for coronary heart disease, myocardial infarction and angina pectoris, type 2 diabetes, triglycerides, total cholesterol and systolic blood pressure served as key mediators, with mediation proportions ranging from 11.76% to 33.33%. Alanine additionally mediated the effect of lower BW on angina pectoris, accounting for 14.29% of the total effect (table 2, figure 4).

**Table 2:** Proportion of the total effect of BW on specific CVDs mediated by metabolic traits

Mediator	Total effect β (95% CI)	The effect of BW on mediator β1 (95% CI)	The effect of mediator on outcome β2 (95% CI)	Mediated proportion (%) (95% CI)
BW on aortic aneurysm	0.38 (0.19 to 0.56)			
Appendicular lean mass		0.47 (0.37 to 0.57)	0.22 (0.11 to 0.32)	26.32% (13.16% to 42.11%)
BMI		0.11 (0.05 to 0.17)	0.40 (0.28 to 0.53)	10.53% (5.26% to 18.42%)
BW on venous thromboembolism	0.19 (0.08 to 0.31)			
Height		0.60 (0.38 to 0.82)	0.22 (0.16 to 0.27)	68.42% (42.11% to 100.00%)
Appendicular lean mass		0.47 (0.37 to 0.57)	0.18 (0.11 to 0.25)	42.11% (26.32% to 68.42%)
BMI		0.11 (0.05 to 0.17)	0.36 (0.28 to 0.44)	21.05% (10.53% to 31.58%)
Systolic blood pressure		−2.12 (−3.21 to −1.03)	−0.01 (−0.02 to −0.01)	15.79% (5.26% to 21.05%)
BW on atrial fibrillation	0.29 (0.19 to 0.39)			
Height		0.60 (0.38 to 0.82)	0.29 (0.24 to 0.34)	58.62% (37.93% to 86.21%)
Appendicular lean mass		0.47 (0.37 to 0.57)	0.29 (0.22 to 0.36)	48.28% (34.48% to 62.07%)
BMI		0.11 (0.05 to 0.17)	0.30 (0.23 to 0.37)	10.34% (3.45% to 17.24%)
BW on coronary heart disease	−0.32 (−0.43 to −0.22)			
Type 2 diabetes		−0.56 (−0.74 to −0.37)	0.15 (0.10 to 0.19)	25.00% (15.62% to 37.50%)
Height		0.60 (0.38 to 0.82)	−0.11 (−0.17 to −0.06)	21.88% (9.38% to 34.38%)
Systolic blood pressure		−2.12 (−3.21 to −1.03)	0.03 (0.02 to 0.04)	21.88% (9.38% to 31.25%)
Triglycerides		−0.14 (−0.19 to −0.09)	0.36 (0.26 to 0.47)	15.62% (9.38% to 25.00%)
Total cholesterol		−0.09 (−0.15 to −0.04)	0.47 (0.38 to 0.56)	12.50% (6.25% to 21.88%)
BW on myocardial infarction	−0.34 (−0.46 to −0.22)			
Type 2 diabetes		−0.56 (−0.74 to −0.37)	0.14 (0.09 to 0.19)	23.53% (11.76% to 35.29%)
Systolic blood pressure		−2.12 (−3.21 to −1.03)	0.03 (0.02 to 0.03)	17.65% (8.82% to 26.47%)
Triglycerides		−0.14 (−0.19 to −0.09)	0.35 (0.25 to 0.45)	14.71% (8.82% to 20.59%)
Total cholesterol		−0.09 (−0.15 to −0.04)	0.43 (0.33 to 0.52)	11.76% (5.88% to 20.59%)
BW on angina pectoris	−0.21 (−0.32 to −0.10)			
Systolic blood pressure		−2.12 (−3.21 to −1.03)	0.04 (0.03 to 0.04)	33.33% (14.29% to 52.38%)
Type 2 diabetes		−0.56 (−0.74 to −0.37)	0.12 (0.07 to 0.17)	33.33% (14.29% to 52.38%)
Triglycerides		−0.14 (−0.19 to −0.09)	0.41 (0.33 to 0.49)	28.57% (19.05% to 38.10%)
Total cholesterol		−0.09 (−0.15 to −0.04)	0.34 (0.25 to 0.42)	14.29% (4.76% to 23.81%)
Alanine		−0.11 (−0.17 to −0.06)	0.26 (0.13 to 0.40)	14.29% (4.76% to 23.81%)

Total effect (β) represents the effect of BW on CVD estimated using the IVW method in two-sample MR analyses. β₁ denotes the effect of BW on each metabolic mediator, also derived from a two-sample IVW MR. β₂ indicates the effect of each mediator on CVD outcomes, obtained from MVMR analyses. The indirect (mediation) effect was calculated as the product of β₁ and β₂ (β₁×β₂). The proportion mediated was computed by dividing the indirect effect by the total effect. SEs were estimated using the delta method.

BMI, body mass index; BW, birth weight; CVD, cardiovascular disease; IVW, inverse-variance weighted ; MR, Mendelian randomisation; MVMR, multivariable Mendelian randomisation.

## Discussion

In this comprehensive MR study, we systematically assessed the causal relationships between BW and a broad spectrum of CVD and quantified the mediating roles of metabolic traits. Our findings revealed distinct causal pathways: genetically predicted lower BW increased risks of coronary heart disease, myocardial infarction and angina pectoris, mediated mainly by type 2 diabetes, systolic blood pressure, total cholesterol and triglycerides. Conversely, higher BW was associated with elevated risks of aortic aneurysm, venous thromboembolism and atrial fibrillation, primarily mediated through body composition traits such as height, BMI and appendicular lean mass.

The observed associations support the DOHaD hypothesis, suggesting that adverse intrauterine conditions resulting in restricted fetal growth can lead to long-term cardiovascular risk through lasting metabolic and physiological adaptations.^19^ Previous epidemiological and genetic studies have consistently demonstrated an inverse relationship between lower BW and coronary diseases, while higher BW has been linked with atrial fibrillation risk.^11 20^ Our findings expand on existing literature by systematically examining a wider range of CVD outcomes and quantitatively assessing the role of specific metabolic mediators. Our study provides novel evidence linking higher BW to increased risks of aortic aneurysm and venous thromboembolism. The mediation analyses identified BMI and appendicular lean mass as critical pathways. Prior research has indicated that larger body size and increased muscle mass may contribute to greater susceptibility to aneurysm through structural alterations in the aorta.^21 22^ Similarly, increased height, BMI and lean mass were implicated in heightened venous thromboembolism risk, consistent with existing evidence on the relationship between larger body size and thrombogenic potential, possibly due to altered haemodynamic conditions and increased venous stasis.^23^,^25^

The association between higher BW and elevated atrial fibrillation risk was mediated significantly by height and lean mass. Previous studies have suggested that increased stature and lean body mass could contribute to cardiac chamber enlargement and elevated atrial pressure, ultimately predisposing individuals to atrial fibrillation through atrial structural remodelling.^26^,^28^ Our findings reinforce these observations, highlighting how early developmental factors can influence later cardiac structure and function. For coronary heart disease, myocardial infarction and angina pectoris, our analysis confirmed type 2 diabetes, triglycerides, total cholesterol and systolic blood pressure as key mediators linking lower BW to elevated disease risk. These metabolic disturbances are likely due to impaired pancreatic β-cell development,^29^ lipid dysregulation^30^ and vascular changes^31^ associated with lower BW and subsequent metabolic catch-up growth. In addition, we found that alanine mediated the effect of lower BW on angina pectoris, which may be explained by amino acid imbalance-induced endothelial dysfunction, ultimately contributing to coronary artery narrowing.^32^

Our findings are primarily based on GWAS data from individuals of European ancestry. Although genetic architecture and allele frequencies vary across populations, the DOHaD hypothesis suggests that the biological mechanisms linking fetal growth to cardiovascular risk are likely to be broadly relevant.^33^ Thus, the implications of our study for cardiovascular risk worldwide remain significant. Specifically, our results underscore the importance of early-life interventions, as BW influences lifelong cardiovascular risk through metabolic pathways.^34^ As a simple and accessible marker, BW can help identify high-risk populations and guide targeted preventive strategies, particularly in resource-limited settings.^35^ Moreover, our study highlights key metabolic traits, such as glucose metabolism, lipid regulation and blood pressure, that mediate the relationship between BW and CVDs, providing mechanistic insights for developing precision prevention strategies. While genetic and environmental contexts differ across ancestries, pathological mechanisms and disease progression may be comparable, reinforcing the need for global health policies that incorporate early-life factors into CVD prevention.^36 37^ This is especially critical in regions with high rates of undernutrition or overnutrition, where prenatal and early-life interventions may substantially reduce the global burden of CVD.^38^

Our study has several notable strengths, including the application of robust MR methodologies, the use of large-scale GWAS datasets and the implementation of extensive sensitivity analyses, all of which enhance the reliability and causal interpretation of the findings. Nonetheless, several limitations should be acknowledged. First, our two-sample MR framework estimates an average linear effect of genetically predicted BW and does not permit formal testing of non-linear or U-shaped associations. Future studies employing non-linear MR methods and individual-level data will be important to examine such potential relationships more rigorously. Second, the GWAS datasets used were predominantly derived from individuals of European ancestry, which may limit the generalisability of our findings to non-European populations. Finally, although we conducted multiple sensitivity analyses to mitigate the impact of horizontal pleiotropy, the possibility of residual pleiotropic effects cannot be fully excluded.

In conclusion, our results demonstrate that BW influences adult cardiovascular risk through multiple, distinct metabolic pathways, underscoring the importance of optimal fetal growth. These findings highlight critical opportunities for targeted early-life interventions and lifelong metabolic management to mitigate CVD burden.

## Supplementary material

10.1136/openhrt-2025-003561 online supplemental file 1

10.1136/openhrt-2025-003561 online supplemental figure 1

## Data Availability

Data are available in a public, open access repository.
