# Supplementary material for: Impact of birth weight on cardiovascular disease and mediating role of metabolic traits: a Mendelian randomisation study
Source: Open Heart. 2025 Oct 31;12(2):e003561. doi: 10.1136/openhrt-2025-003561 (PMC12581085; doi:10.1136/openhrt-2025-003561)
Supplement: online supplemental file 1 [file openhrt-12-2-s001.docx]

**Supplementary Materials**

[Supplementary Table 1. Sensitivity Analyses of Causal Associations Between Birth Weight and Cardiovascular Diseases in Two-Sample Mendelian Randomization 2](#_Toc199178871)

[Supplementary Table 2. Sensitivity Analyses of Causal Associations Between Birth Weight and Potential Metabolic Mediators in Two-Sample Mendelian Randomization 5](#_Toc199178872)

[Supplementary Table 3. Multivariable Mendelian Randomization (MVMR) Estimates for the Causal Associations of Qualified Metabolic Mediators with Coronary Heart Disease, Myocardial Infarction, Angina Pectoris, Aortic Aneurysm, Venous Thromboembolism, and Atrial Fibrillation, Adjusted for Birth Weight 10](#_Toc199178873)

[Supplementary Figure 1. Leave-one-out analysis for the associations between birth weight and coronary heart disease 21](#_Toc199178874)

[Supplementary Figure 2. Leave-one-out analysis for the associations between birth weight and myocardial infarction 22](#_Toc199178875)

[Supplementary Figure 3. Leave-one-out analysis for the associations between birth weight and angina pectoris 23](#_Toc199178876)

[Supplementary Figure 4. Leave-one-out analysis for the associations between birth weight and ischaemic stroke 24](#_Toc199178877)

[Supplementary Figure 5. Leave-one-out analysis for the associations between birth weight and peripheral artery disease 25](#_Toc199178878)

[Supplementary Figure 6. Leave-one-out analysis for the associations between birth weight and rheumatic heart disease 26](#_Toc199178879)

[Supplementary Figure 7. Leave-one-out analysis for the associations between birth weight and myocarditis 27](#_Toc199178880)

[Supplementary Figure 8. Leave-one-out analysis for the associations between birth weight and pericarditis 28](#_Toc199178881)

[Supplementary Figure 9. Leave-one-out analysis for the associations between birth weight and endocarditis 29](#_Toc199178882)

[Supplementary Figure 10. Leave-one-out analysis for the associations between birth weight and aortic aneurysm 30](#_Toc199178883)

[Supplementary Figure 11. Leave-one-out analysis for the associations between birth weight and heart failure 31](#_Toc199178884)

[Supplementary Figure 12. Leave-one-out analysis for the associations between birth weight and intracerebral haemorrhage 32](#_Toc199178885)

[Supplementary Figure 13. Leave-one-out analysis for the associations between birth weight and subarachnoid haemorrhage 33](#_Toc199178886)

[Supplementary Figure 14. Leave-one-out analysis for the associations between birth weight and venous thromboembolism 34](#_Toc199178887)

[Supplementary Figure 15. Leave-one-out analysis for the associations between birth weight and non-rheumatic valvular disease 35](#_Toc199178888)

[Supplementary Figure 16. Leave-one-out analysis for the associations between birth weight and atrial fibrillation 36](#_Toc199178889)

# Supplementary Table 1. Sensitivity Analyses of Causal Associations Between Birth Weight and Cardiovascular Diseases in Two-Sample Mendelian Randomization

| Exposure | Outcome | No. of SNPs used | F-statistic | P-value for Cochran's Q | MR Method | OR (95% CI) | *P* | MR-Egger intercept (*P* value) |
| --- | --- | --- | --- | --- | --- | --- | --- | --- |
| BW | Coronary heart disease | 149 | 56.71 | 6.23E-09 | Inverse variance weighted | 0.72 (0.65, 0.81) | 4.02E-09 |  |
|  |  | 149 |  |  | MR Egger | 0.71 (0.52, 0.96) | 2.93E-02 | 8.81E-01 |
|  |  | 149 |  |  | Weighted median | 0.75 (0.65, 0.86) | 3.30E-05 |  |
|  |  | 149 |  |  | Weighted mode | 0.78 (0.61, 0.99) | 4.05E-02 |  |
|  |  | 147 |  |  | MR-PRESSO | 0.75 (0.68, 0.83) | 4.40E-08 |  |
| BW | Myocardial infarction | 149 | 56.71 | 3.72E-08 | Inverse variance weighted | 0.71 (0.63, 0.80) | 1.53E-08 |  |
|  |  | 149 |  |  | MR Egger | 0.76 (0.54, 1.07) | 1.17E-01 | 6.69E-01 |
|  |  | 149 |  |  | Weighted median | 0.72 (0.62, 0.84) | 2.76E-05 |  |
|  |  | 149 |  |  | Weighted mode | 0.72 (0.55, 0.95) | 1.89E-02 |  |
|  |  | 147 |  |  | MR-PRESSO | 0.74 (0.67, 0.82) | 1.31E-07 |  |
| BW | Angina pectoris | 149 | 56.78 | 9.94E-21 | Inverse variance weighted | 0.81 (0.72, 0.90) | 1.29E-04 |  |
|  |  | 149 |  |  | MR Egger | 0.95 (0.69, 1.30) | 7.28E-01 | 2.98E-01 |
|  |  | 149 |  |  | Weighted median | 0.85 (0.75, 0.96) | 1.04E-02 |  |
|  |  | 149 |  |  | Weighted mode | 0.74 (0.57, 0.97) | 3.16E-02 |  |
|  |  | 143 |  |  | MR-PRESSO | 0.83 (0.75, 0.91) | 1.27E-04 |  |
| BW | Ischaemic stroke | 150 | 55.08 | 5.71E-14 | Inverse variance weighted | 0.90 (0.79, 1.01) | 8.06E-02 |  |
|  |  | 150 |  |  | MR Egger | 0.96 (0.66, 1.41) | 8.53E-01 | 6.82E-01 |
|  |  | 150 |  |  | Weighted median | 0.88 (0.76, 1.01) | 6.41E-02 |  |
|  |  | 150 |  |  | Weighted mode | 0.82 (0.58, 1.16) | 2.67E-01 |  |
|  |  | 148 |  |  | MR-PRESSO | 0.94 (0.84, 1.05) | 2.53E-01 |  |
| BW | Peripheral artery disease | 145 | 57.09 | 5.49E-07 | Inverse variance weighted | 0.82 (0.71, 0.95) | 9.22E-03 |  |
|  |  | 145 |  |  | MR Egger | 0.89 (0.57, 1.37) | 5.91E-01 | 7.03E-01 |
|  |  | 145 |  |  | Weighted median | 0.91 (0.76, 1.10) | 3.50E-01 |  |
|  |  | 145 |  |  | Weighted mode | 0.93 (0.67, 1.29) | 6.72E-01 |  |
|  |  | 143 |  |  | MR-PRESSO | 0.86 (0.74, 0.99) | 3.56E-02 |  |
| BW | rheumatic heart disease | 149 | 56.78 | 7.51E-01 | Inverse variance weighted | 1.49 (0.59, 3.79) | 4.02E-01 |  |
|  |  | 149 |  |  | MR Egger | 0.92 (0.06, 13.53) | 9.49E-01 | 7.06E-01 |
|  |  | 149 |  |  | Weighted median | 1.53 (0.36, 6.45) | 5.60E-01 |  |
|  |  | 149 |  |  | Weighted mode | 1.84 (0.10, 34.69) | 6.84E-01 |  |
|  |  | 149 |  |  | MR-PRESSO | 1.49 (0.61, 3.64) | 3.82E-01 |  |
| BW | Myocarditis | 149 | 56.78 | 6.43E-01 | Inverse variance weighted | 0.94 (0.71, 1.25) | 6.71E-01 |  |
|  |  | 149 |  |  | MR Egger | 4.08 (1.83, 9.10) | 7.79E-04 | 1.96E-04 |
|  |  | 149 |  |  | Weighted median | 1.29 (0.85, 1.96) | 2.32E-01 |  |
|  |  | 149 |  |  | Weighted mode | 1.98 (0.96, 4.07) | 6.67E-02 |  |
|  |  | 149 |  |  | MR-PRESSO | 0.94 (0.71, 1.25) | 6.72E-01 |  |
| BW | Pericarditis | 149 | 56.78 | 7.23E-01 | Inverse variance weighted | 0.90 (0.64, 1.26) | 5.29E-01 |  |
|  |  | 149 |  |  | MR Egger | 1.43 (0.53, 3.84) | 4.75E-01 | 3.21E-01 |
|  |  | 149 |  |  | Weighted median | 1.12 (0.67, 1.85) | 6.70E-01 |  |
|  |  | 149 |  |  | Weighted mode | 1.10 (0.40, 3.09) | 8.49E-01 |  |
|  |  | 149 |  |  | MR-PRESSO | 0.90 (0.64, 1.25) | 5.15E-01 |  |
| BW | Endocarditis | 149 | 56.78 | 8.08E-01 | Inverse variance weighted | 0.89 (0.62, 1.26) | 5.02E-01 |  |
|  |  | 149 |  |  | MR Egger | 0.89 (0.32, 2.44) | 8.15E-01 | 9.99E-01 |
|  |  | 149 |  |  | Weighted median | 0.83 (0.49, 1.42) | 5.06E-01 |  |
|  |  | 149 |  |  | Weighted mode | 0.44 (0.09, 2.13) | 3.12E-01 |  |
|  |  | 149 |  |  | MR-PRESSO | 0.89 (0.64, 1.24) | 4.78E-01 |  |
| BW | Aortic aneurysm | 149 | 56.78 | 1.84E-12 | Inverse variance weighted | 1.46 (1.21, 1.75) | 7.03E-05 |  |
|  |  | 149 |  |  | MR Egger | 1.89 (1.11, 3.21) | 2.08E-02 | 3.11E-01 |
|  |  | 149 |  |  | Weighted median | 1.39 (1.12, 1.72) | 2.62E-03 |  |
|  |  | 149 |  |  | Weighted mode | 1.40 (0.93, 2.11) | 1.10E-01 |  |
|  |  | 146 |  |  | MR-PRESSO | 1.40 (1.18, 1.67) | 2.36E-04 |  |
| BW | Heart failure | 149 | 56.78 | 8.44E-09 | Inverse variance weighted | 1.07 (0.97, 1.18) | 2.06E-01 |  |
|  |  | 149 |  |  | MR Egger | 1.29 (0.97, 1.72) | 8.17E-02 | 1.64E-01 |
|  |  | 149 |  |  | Weighted median | 1.07 (0.95, 1.21) | 2.55E-01 |  |
|  |  | 149 |  |  | Weighted mode | 1.13 (0.92, 1.38) | 2.61E-01 |  |
|  |  | 148 |  |  | MR-PRESSO | 1.08 (0.98, 1.19) | 1.08E-01 |  |
| BW | Intracerebral haemorrhage | 149 | 56.78 | 4.42E-02 | Inverse variance weighted | 0.96 (0.79, 1.17) | 7.02E-01 |  |
|  |  | 149 |  |  | MR Egger | 0.88 (0.50, 1.54) | 6.55E-01 | 7.37E-01 |
|  |  | 149 |  |  | Weighted median | 1.04 (0.79, 1.38) | 7.76E-01 |  |
|  |  | 149 |  |  | Weighted mode | 1.18 (0.49, 2.82) | 7.12E-01 |  |
|  |  | 149 |  |  | MR-PRESSO | 0.96 (0.79, 1.17) | 7.03E-01 |  |
| BW | Subarachnoid haemorrhage | 149 | 56.78 | 2.31E-03 | Inverse variance weighted | 0.94 (0.75, 1.18) | 5.97E-01 |  |
|  |  | 149 |  |  | MR Egger | 1.39 (0.72, 2.67) | 3.26E-01 | 2.16E-01 |
|  |  | 149 |  |  | Weighted median | 1.01 (0.75, 1.38) | 9.27E-01 |  |
|  |  | 149 |  |  | Weighted mode | 0.96 (0.43, 2.10) | 9.10E-01 |  |
|  |  | 148 |  |  | MR-PRESSO | 0.97 (0.78, 1.20) | 7.84E-01 |  |
| BW | Venous thromboembolism | 149 | 56.78 | 1.90E-11 | Inverse variance weighted | 1.22 (1.09, 1.36) | 7.38E-04 |  |
|  |  | 149 |  |  | MR Egger | 1.50 (1.09, 2.08) | 1.52E-02 | 1.74E-01 |
|  |  | 149 |  |  | Weighted median | 1.18 (1.04, 1.34) | 1.12E-02 |  |
|  |  | 149 |  |  | Weighted mode | 1.11 (0.82, 1.51) | 5.00E-01 |  |
|  |  | 145 |  |  | MR-PRESSO | 1.19 (1.08, 1.32) | 7.71E-04 |  |
| BW | Non-rheumatic valvular disease | 149 | 56.78 | 3.23E-09 | Inverse variance weighted | 1.10 (0.98, 1.23) | 9.44E-02 |  |
|  |  | 149 |  |  | MR Egger | 1.34 (0.97, 1.84) | 7.62E-02 | 2.01E-01 |
|  |  | 149 |  |  | Weighted median | 1.06 (0.93, 1.21) | 4.01E-01 |  |
|  |  | 149 |  |  | Weighted mode | 0.96 (0.63, 1.47) | 8.65E-01 |  |
|  |  | 148 |  |  | MR-PRESSO | 1.11 (1.00, 1.24) | 6.33E-02 |  |
| BW | Atrial fibrillation | 134 | 57.61 | 2.25E-15 | Inverse variance weighted | 1.34 (1.21, 1.48) | 1.93E-08 |  |
|  |  | 134 |  |  | MR Egger | 1.57 (1.19, 2.07) | 1.87E-03 | 2.26E-01 |
|  |  | 134 |  |  | Weighted median | 1.41 (1.26, 1.57) | 1.80E-09 |  |
|  |  | 134 |  |  | Weighted mode | 1.55 (1.20, 1.99) | 1.02E-03 |  |
|  |  | 129 |  |  | MR-PRESSO | 1.33 (1.21, 1.45) | 9.18E-09 |  |

**Abbreviations:** BW, birth weight

# Supplementary Table 2. Sensitivity Analyses of Causal Associations Between Birth Weight and Potential Metabolic Mediators in Two-Sample Mendelian Randomization

| Exposure | Mediator | No. of SNPs used | F-statistic | *P* value for Cochran's Q | MR Method | β (95% CI) | OR (95% CI) | *P value* | MR-Egger intercept (*P* value) |
| --- | --- | --- | --- | --- | --- | --- | --- | --- | --- |
| BW | Body mass index | 63 | 59.43 | 2.77E-74 | Inverse variance weighted^a^ | 0.11 (0.05, 0.17) |  | 4.92E-04 |  |
|  |  | 63 |  |  | MR Egger | 0.10 (-0.09, 0.28) |  | 3.14E-01 | 9.03E-01 |
|  |  | 63 |  |  | Weighted median | 0.07 (0.03, 0.11) |  | 1.18E-03 |  |
|  |  | 63 |  |  | Weighted mode | 0.06 (0.01, 0.11) |  | 2.26E-02 |  |
|  |  | 52 |  |  | MR-PRESSO | 0.07 (0.04, 0.11) |  | 3.26E-04 |  |
|  |  | 62 | 59.88 | 8.20E-63 | Inverse variance weighted^b^ | 0.10 (0.04, 0.16) |  | 6.47E-04 |  |
|  | Height | 47 | 61.94 | 0.00E+00 | Inverse variance weighted^a^ | 0.60 (0.38, 0.82) |  | 7.39E-08 |  |
|  |  | 47 |  |  | MR Egger | 0.42 (-0.29, 1.14) |  | 2.54E-01 | 6.14E-01 |
|  |  | 47 |  |  | Weighted median | 0.10 (0.05, 0.14) |  | 1.75E-05 |  |
|  |  | 47 |  |  | Weighted mode | 0.11 (0.07, 0.14) |  | 3.81E-07 |  |
|  |  | 17 |  |  | MR-PRESSO | 0.58 (0.48, 0.68) |  | 4.99E-09 |  |
|  |  | 43 | 60.47 | 0.00E+00 | Inverse variance weighted^b^ | 0.65 (0.43, 0.88) |  | 1.36E-08 |  |
|  | Appendicular  lean mass | 151 | 56.5 | 0.00E+00 | Inverse variance weighted^a^ | 0.47 (0.37, 0.57) |  | 1.51E-20 |  |
|  |  | 151 |  |  | MR Egger | 0.38 (0.11, 0.65) |  | 6.09E-03 | 4.74E-01 |
|  |  | 151 |  |  | Weighted median | 0.17 (0.13, 0.22) |  | 2.55E-14 |  |
|  |  | 151 |  |  | Weighted mode | 0.14 (0.09, 0.19) |  | 3.32E-07 |  |
|  |  | 151 |  |  | MR-PRESSO | 0.47 (0.37, 0.57) |  | 1.65E-16 |  |
|  |  | 142 | 56.52 | 0.00E+00 | Inverse variance weighted^b^ | 0.41 (0.31, 0.50) |  | 2.76E-17 |  |
|  | Type 2  diabetes | 152 | 56.34 | 1.88E-308 | Inverse variance weighted^a^ | -0.56 (-0.74, -0.37) | 0.57 (0.48, 0.69) | 9.16E-09 |  |
|  |  | 152 |  |  | MR Egger | -1.15 (-1.66, -0.64) | 0.32 (0.19, 0.53) | 1.97E-05 | 1.57E-02 |
|  |  | 152 |  |  | Weighted median | -0.36 (-0.47, -0.25) | 0.70 (0.63, 0.78) | 1.61E-10 |  |
|  |  | 152 |  |  | Weighted mode | -0.38 (-0.58, -0.17) | 0.68 (0.56, 0.84) | 4.32E-04 |  |
|  |  | 132 |  |  | MR-PRESSO | -0.43 (-0.52, -0.33) | 0.65 (0.59, 0.72) | 1.93E-14 |  |
|  |  | 149 | 55.56 | 6.67E-196 | Inverse variance weighted^b^ | -0.52 (-0.68, -0.36) | 0.59 (0.51, 0.70) | 4.04E-10 |  |
|  | Fasting  glucose | 151 | 56.46 | 0.00E+00 | Inverse variance weighted^a^ | -0.06 (-0.12, 0.00) |  | 6.92E-02 |  |
|  |  | 151 |  |  | MR Egger | -0.21 (-0.37, -0.04) |  | 1.71E-02 | 6.17E-02 |
|  |  | 151 |  |  | Weighted median | -0.05 (-0.08, -0.02) |  | 7.93E-04 |  |
|  |  | 151 |  |  | Weighted mode | -0.03 (-0.08, 0.02) |  | 2.82E-01 |  |
|  |  | 141 |  |  | MR-PRESSO | -0.06 (-0.08, -0.03) |  | 3.86E-06 |  |
|  |  | 150 | 55.59 | 0.00E+00 | Inverse variance weighted^b^ | -0.04 (-0.10, 0.02) |  | 1.84E-01 |  |
|  | Two hours  glucose | 151 | 56.46 | 1.90E-17 | Inverse variance weighted^a^ | -0.23 (-0.33, -0.12) |  | 2.73E-05 |  |
|  |  | 151 |  |  | MR Egger | -0.34 (-0.63, -0.05) |  | 2.43E-02 | 4.23E-01 |
|  |  | 151 |  |  | Weighted median | -0.23 (-0.35, -0.11) |  | 2.43E-04 |  |
|  |  | 151 |  |  | Weighted mode | -0.24 (-0.75, 0.27) |  | 3.57E-01 |  |
|  |  | 147 |  |  | MR-PRESSO | -0.22 (-0.31, -0.14) |  | 1.28E-06 |  |
|  |  | 150 | 55.59 | 2.22E-09 | Inverse variance weighted^b^ | -0.18 (-0.28, -0.08) |  | 2.58E-04 |  |
|  | Total  cholesterol | 152 | 56.46 | 0.00E+00 | Inverse variance weighted^a^ | -0.09 (-0.15, -0.04) |  | 9.60E-04 |  |
|  |  | 152 |  |  | MR Egger | -0.07 (-0.22, 0.09) |  | 3.88E-01 | 7.27E-01 |
|  |  | 152 |  |  | Weighted median | -0.04 (-0.06, -0.01) |  | 2.41E-03 |  |
|  |  | 152 |  |  | Weighted mode | -0.03 (-0.06, 0.00) |  | 5.24E-02 |  |
|  |  | 152 |  |  | MR-PRESSO | -0.09 (-0.15, -0.04) |  | 1.20E-03 |  |
|  |  | 150 | 56.19 | 0.00E+00 | Inverse variance weighted^b^ | -0.10 (-0.15, -0.05) |  | 1.44E-04 |  |
|  | High-density  lipoprotein | 152 | 56.46 | 0.00E+00 | Inverse variance weighted^a^ | 0.05 (0.00, 0.09) |  | 3.22E-02 |  |
|  |  | 152 |  |  | MR Egger | -0.05 (-0.17, 0.06) |  | 3.83E-01 | 7.77E-02 |
|  |  | 152 |  |  | Weighted median | 0.04 (0.01, 0.06) |  | 1.73E-03 |  |
|  |  | 152 |  |  | Weighted mode | 0.06 (0.01, 0.11) |  | 3.17E-02 |  |
|  |  | 126 |  |  | MR-PRESSO | 0.04 (0.02, 0.06) |  | 1.03E-03 |  |
|  |  | 152 | 56.34 | 0.00E+00 | Inverse variance weighted^b^ | 0.05 (0.00, 0.09) |  | 3.22E-02 |  |
|  | Low-density  lipoprotein | 152 | 56.46 | 0.00E+00 | Inverse variance weighted^a^ | -0.07 (-0.13, -0.02) |  | 6.49E-03 |  |
|  |  | 152 |  |  | MR Egger | -0.04 (-0.19, 0.10) |  | 5.76E-01 | 6.51E-01 |
|  |  | 152 |  |  | Weighted median | -0.03 (-0.05, -0.01) |  | 6.50E-03 |  |
|  |  | 152 |  |  | Weighted mode | -0.01 (-0.05, 0.02) |  | 3.95E-01 |  |
|  |  | 152 |  |  | MR-PRESSO | -0.07 (-0.13, -0.02) |  | 7.26E-03 |  |
|  |  | 151 | 56.18 | 0.00E+00 | Inverse variance weighted^b^ | -0.08 (-0.13, -0.03) |  | 1.12E-03 |  |
|  | Triglycerides | 152 | 56.46 | 0.00E+00 | Inverse variance weighted^a^ | -0.14 (-0.19, -0.09) |  | 5.38E-09 |  |
|  |  | 152 |  |  | MR Egger | -0.07 (-0.20, 0.06) |  | 3.21E-01 | 2.32E-01 |
|  |  | 152 |  |  | Weighted median | -0.06 (-0.09, -0.04) |  | 2.49E-06 |  |
|  |  | 152 |  |  | Weighted mode | -0.01 (-0.05, 0.03) |  | 6.08E-01 |  |
|  |  | 116 |  |  | MR-PRESSO | -0.13 (-0.15, -0.10) |  | 6.89E-17 |  |
|  |  | 151 | 56.36 | 0.00E+00 | Inverse variance weighted^b^ | -0.13 (-0.18, -0.09) |  | 6.68E-09 |  |
|  | Hypertension | 149 | 56.78 | 1.41E-134 | Inverse variance weighted^a^ | -0.11 (-0.22, 0.01) | 0.90 (0.80, 1.01) | 7.30E-02 |  |
|  |  | 149 |  |  | MR Egger | 0.23 (-0.11, 0.56) | 1.25 (0.90, 1.75) | 1.88E-01 | 3.92E-02 |
|  |  | 149 |  |  | Weighted median | -0.05 (-0.14, 0.03) | 0.95 (0.87, 1.04) | 2.41E-01 |  |
|  |  | 149 |  |  | Weighted mode | -0.02 (-0.21, 0.17) | 0.98 (0.81, 1.19) | 8.55E-01 |  |
|  |  | 135 |  |  | MR-PRESSO | -0.14 (-0.22, -0.06) | 0.87 (0.80, 0.94) | 7.63E-04 |  |
|  |  | 149 | 56.78 | 1.41E-134 | Inverse variance weighted^b^ | -0.11 (-0.22, 0.01) | 0.90 (0.80, 1.01) | 7.30E-02 |  |
|  | Systolic blood  pressure | 135 | 55.80 | 0.00E+00 | Inverse variance weighted^a^ | -2.12 (-3.21, -1.03) |  | 1.41E-04 |  |
|  |  | 135 |  |  | MR Egger | 0.57 (-2.66, 3.79) |  | 7.30E-01 | 8.52E-02 |
|  |  | 135 |  |  | Weighted median | -0.75 (-1.38, -0.12) |  | 1.94E-02 |  |
|  |  | 135 |  |  | Weighted mode | 0.50 (-0.89, 1.89) |  | 4.79E-01 |  |
|  |  | 100 |  |  | MR-PRESSO | -1.81 (-2.46, -1.16) |  | 4.04E-07 |  |
|  |  | 135 | 55.80 | 0.00E+00 | Inverse variance weighted^b^ | -2.12 (-3.21, -1.03) |  | 1.41E-04 |  |
|  | Diastolic blood  pressure | 135 | 55.80 | 0.00E+00 | Inverse variance weighted^a^ | -0.85 (-1.49, -0.21) |  | 9.64E-03 |  |
|  |  | 135 |  |  | MR Egger | 0.53 (-1.36, 2.43) |  | 5.81E-01 | 1.32E-01 |
|  |  | 135 |  |  | Weighted median | -0.42 (-0.79, -0.05) |  | 2.59E-02 |  |
|  |  | 135 |  |  | Weighted mode | 0.60 (-0.43, 1.62) |  | 2.56E-01 |  |
|  |  | 107 |  |  | MR-PRESSO | -0.78 (-1.12, -0.44) |  | 1.64E-05 |  |
|  |  | 134 | 54.72 | 0.00E+00 | Inverse variance weighted^b^ | -0.79 (-1.44, -0.14) |  | 1.72E-02 |  |
|  | Omega3 | 152 | 56.34 | 3.98E-22 | Inverse variance weighted^a^ | -0.07 (-0.13, -0.02) |  | 7.42E-03 |  |
|  |  | 152 |  |  | MR Egger | -0.07 (-0.21, 0.08) |  | 3.72E-01 | 9.18E-01 |
|  |  | 152 |  |  | Weighted median | -0.02 (-0.08, 0.04) |  | 4.83E-01 |  |
|  |  | 152 |  |  | Weighted mode | -0.02 (-0.18, 0.13) |  | 7.94E-01 |  |
|  |  | 145 |  |  | MR-PRESSO | -0.04 (-0.08, -0.00) |  | 3.91E-02 |  |
|  |  | 152 | 56.34 | 3.98E-22 | Inverse variance weighted^b^ | -0.07 (-0.13, -0.02) |  | 7.42E-03 |  |
|  | Omega6 | 152 | 56.34 | 1.32E-29 | Inverse variance weighted^a^ | -0.10 (-0.15, -0.04) |  | 8.59E-04 |  |
|  |  | 152 |  |  | MR Egger | -0.15 (-0.30, 0.01) |  | 6.34E-02 | 4.95E-01 |
|  |  | 152 |  |  | Weighted median | -0.08 (-0.13, -0.02) |  | 1.00E-02 |  |
|  |  | 152 |  |  | Weighted mode | -0.05 (-0.15, 0.05) |  | 3.46E-01 |  |
|  |  | 146 |  |  | MR-PRESSO | -0.09 (-0.13, -0.04) |  | 1.21E-04 |  |
|  |  | 152 | 56.34 | 1.32E-29 | Inverse variance weighted^b^ | -0.10 (-0.15, -0.04) |  | 8.59E-04 |  |
|  | Docosahexaenoic  acid | 152 | 56.34 | 2.74E-09 | Inverse variance weighted^a^ | -0.03 (-0.07, 0.02) |  | 2.26E-01 |  |
|  |  | 152 |  |  | MR Egger | -0.10 (-0.22, 0.02) |  | 1.13E-01 | 2.20E-01 |
|  |  | 152 |  |  | Weighted median | -0.02 (-0.07, 0.04) |  | 5.05E-01 |  |
|  |  | 152 |  |  | Weighted mode | -0.00 (-0.10, 0.09) |  | 9.29E-01 |  |
|  |  | 149 |  |  | MR-PRESSO | -0.01 (-0.05, 0.03) |  | 6.73E-01 |  |
|  |  | 152 | 56.34 | 2.74E-09 | Inverse variance weighted^b^ | -0.03 (-0.07, 0.02) |  | 2.26E-01 |  |
|  | Linoleic acid | 152 | 56.34 | 2.74E-09 | Inverse variance weighted^a^ | -0.03 (-0.07, 0.02) |  | 2.26E-01 |  |
|  |  | 152 |  |  | MR Egger | -0.10 (-0.22, 0.02) |  | 1.13E-01 | 2.20E-01 |
|  |  | 152 |  |  | Weighted median | -0.02 (-0.07, 0.04) |  | 5.05E-01 |  |
|  |  | 152 |  |  | Weighted mode | -0.00 (-0.10, 0.09) |  | 9.29E-01 |  |
|  |  | 149 |  |  | MR-PRESSO | -0.01 (-0.05, 0.03) |  | 6.73E-01 |  |
|  |  | 152 | 56.34 | 2.74E-09 | Inverse variance weighted^b^ | -0.03 (-0.07, 0.02) |  | 2.26E-01 |  |
|  | Isoleucine | 152 | 56.34 | 9.58E-07 | Inverse variance weighted^a^ | -0.09 (-0.13, -0.04) |  | 7.46E-05 |  |
|  |  | 152 |  |  | MR Egger | -0.09 (-0.20, 0.03) |  | 1.32E-01 | 9.57E-01 |
|  |  | 152 |  |  | Weighted median | -0.10 (-0.15, -0.05) |  | 2.34E-04 |  |
|  |  | 152 |  |  | Weighted mode | -0.10 (-0.20, -0.00) |  | 4.93E-02 |  |
|  |  | 150 |  |  | MR-PRESSO | -0.09 (-0.13, -0.05) |  | 2.72E-05 |  |
|  |  | 152 | 56.34 | 9.58E-07 | Inverse variance weighted^b^ | -0.09 (-0.13, -0.04) |  | 7.46E-05 |  |
|  | Leucine | 152 | 56.34 | 5.98E-13 | Inverse variance weighted^a^ | -0.10 (-0.15, -0.05) |  | 1.94E-05 |  |
|  |  | 152 |  |  | MR Egger | -0.12 (-0.24, 0.01) |  | 6.72E-02 | 7.77E-01 |
|  |  | 152 |  |  | Weighted median | -0.06 (-0.12, -0.01) |  | 1.79E-02 |  |
|  |  | 152 |  |  | Weighted mode | -0.01 (-0.13, 0.10) |  | 8.29E-01 |  |
|  |  | 148 |  |  | MR-PRESSO | -0.10 (-0.14, -0.06) |  | 3.96E-06 |  |
|  |  | 152 | 56.34 | 5.98E-13 | Inverse variance weighted^b^ | -0.10 (-0.15, -0.05) |  | 1.94E-05 |  |
|  | Valine | 152 | 56.34 | 9.17E-24 | Inverse variance weighted^a^ | -0.09 (-0.14, -0.04) |  | 7.69E-04 |  |
|  |  | 152 |  |  | MR Egger | -0.11 (-0.25, 0.04) |  | 1.53E-01 | 8.35E-01 |
|  |  | 152 |  |  | Weighted median | -0.09 (-0.15, -0.04) |  | 6.97E-04 |  |
|  |  | 152 |  |  | Weighted mode | 0.01 (-0.11, 0.12) |  | 8.85E-01 |  |
|  |  | 145 |  |  | MR-PRESSO | -0.11 (-0.16, -0.07) |  | 9.97E-07 |  |
|  |  | 152 | 56.34 | 9.17E-24 | Inverse variance weighted^b^ | -0.09 (-0.14, -0.04) |  | 7.69E-04 |  |
|  | Phenylalanine | 152 | 56.34 | 8.62E-05 | Inverse variance weighted^a^ | -0.08 (-0.12, -0.04) |  | 2.29E-04 |  |
|  |  | 152 |  |  | MR Egger | -0.06 (-0.18, 0.05) |  | 2.61E-01 | 8.02E-01 |
|  |  | 152 |  |  | Weighted median | -0.07 (-0.12, -0.01) |  | 1.47E-02 |  |
|  |  | 152 |  |  | Weighted mode | -0.07 (-0.18, 0.04) |  | 2.28E-01 |  |
|  |  | 150 |  |  | MR-PRESSO | -0.08 (-0.12, -0.04) |  | 9.95E-05 |  |
|  |  | 152 | 56.34 | 8.62E-05 | Inverse variance weighted^b^ | -0.08 (-0.12, -0.04) |  | 2.29E-04 |  |
|  | Tyrosine | 152 | 56.34 | 1.74E-10 | Inverse variance weighted^a^ | -0.09 (-0.14, -0.05) |  | 1.07E-04 |  |
|  |  | 152 |  |  | MR Egger | -0.08 (-0.21, 0.04) |  | 2.00E-01 | 8.75E-01 |
|  |  | 152 |  |  | Weighted median | -0.08 (-0.14, -0.02) |  | 1.01E-02 |  |
|  |  | 152 |  |  | Weighted mode | -0.08 (-0.20, 0.04) |  | 1.78E-01 |  |
|  |  | 151 |  |  | MR-PRESSO | -0.09 (-0.13, -0.04) |  | 2.23E-04 |  |
|  |  | 152 | 56.34 | 1.74E-10 | Inverse variance weighted^b^ | -0.09 (-0.14, -0.05) |  | 1.07E-04 |  |
|  | Alanine | 152 | 56.34 | 5.10E-25 | Inverse variance weighted^a^ | -0.11 (-0.17, -0.06) |  | 8.23E-05 |  |
|  |  | 152 |  |  | MR Egger | -0.17 (-0.32, -0.02) |  | 2.60E-02 | 3.97E-01 |
|  |  | 152 |  |  | Weighted median | -0.05 (-0.10, 0.01) |  | 1.12E-01 |  |
|  |  | 152 |  |  | Weighted mode | 0.03 (-0.08, 0.14) |  | 5.80E-01 |  |
|  |  | 147 |  |  | MR-PRESSO | -0.08 (-0.13, -0.04) |  | 4.32E-04 |  |
|  |  | 151 | 56.44 | 5.24E-20 | Inverse variance weighted^b^ | -0.12 (-0.17, -0.07) |  | 1.12E-05 |  |
|  | Glycine | 152 | 56.34 | 4.71E-11 | Inverse variance weighted^a^ | 0.10 (0.05, 0.14) |  | 2.57E-05 |  |
|  |  | 152 |  |  | MR Egger | 0.06 (-0.06, 0.18) |  | 3.26E-01 | 5.39E-01 |
|  |  | 152 |  |  | Weighted median | 0.05 (-0.00, 0.11) |  | 5.67E-02 |  |
|  |  | 152 |  |  | Weighted mode | 0.03 (-0.09, 0.15) |  | 6.08E-01 |  |
|  |  | 150 |  |  | MR-PRESSO | 0.10 (0.06, 0.14) |  | 5.94E-06 |  |
|  |  | 152 | 56.34 | 4.71E-11 | Inverse variance weighted^b^ | 0.10 (0.05, 0.14) |  | 2.57E-05 |  |

^a^The inverse variance weighted method was performed without excluding overlapping SNPs between BW and mediators in two-sample MR analysis.
^b^The inverse variance weighted method was performed after excluding overlapping SNPs between BW and mediators in two-sample MR analysis.

**Abbreviations:** BW, birth weight; MR, mendelian randomization

# Supplementary Table 3. Multivariable Mendelian Randomization (MVMR) Estimates for the Causal Associations of Qualified Metabolic Mediators with Coronary Heart Disease, Myocardial Infarction, Angina Pectoris, Aortic Aneurysm, Venous Thromboembolism, and Atrial Fibrillation, Adjusted for Birth Weight

| Mediator | Outcome | No. of SNPs used | F-statistic | *P* value for Cochran's Q | MR Method | *β* (95% CI) | OR (95% CI) | *P value* | MR-Egger intercept (P value) |
| --- | --- | --- | --- | --- | --- | --- | --- | --- | --- |
| Body mass index | Coronary  heart disease | 447 | 73.11 | 2.49E-12 | MVMR-IVW^a^ | 0.47 (0.40, 0.55) | 1.61 (1.49, 1.74) | **1.37E-32** |  |
|  |  | 447 |  |  | MVMR-Egger | 0.67 (0.46, 0.89) | 1.96 (1.59, 2.42) | 4.48E-10 | 4.63E-02 |
|  |  | 447 |  |  | MVMR- weighted median | 0.46 (0.36, 0.56) | 1.58 (1.44, 1.74) | 2.72E-20 |  |
|  |  | 446 | 73.10 | 2.00E-12 | MVMR-IVW^b^ | 0.47 (0.40, 0.55) | 1.61 (1.49, 1.74) | 1.62E-32 |  |
| Height |  | 965 | 250.49 | 9.18E-39 | MVMR-IVW^a^ | -0.11 (-0.17, -0.06) | 0.89 (0.85, 0.94) | **6.79E-05** |  |
|  |  | 965 |  |  | MVMR-Egger | -0.14 (-0.24, -0.04) | 0.87 (0.79, 0.96) | 4.67E-03 | 4.96E-01 |
|  |  | 965 |  |  | MVMR- weighted median | -0.08 (-0.14, -0.01) | 0.93 (0.87, 0.99) | 1.97E-02 |  |
|  |  | 961 | 250.54 | 3.44E-39 | MVMR-IVW^b^ | -0.11 (-0.17, -0.06) | 0.89 (0.85, 0.95) | 9.71E-05 |  |
| Appendicular  lean mass |  | 598 | 7.68 | 1.53E-29 | MVMR-IVW^a^ | -0.09 (-0.15, -0.02) | 0.92 (0.86, 0.98) | 1.55E-02 |  |
|  |  | 598 |  |  | MVMR-Egger | -0.09 (-0.16, -0.02) | 0.91 (0.85, 0.98) | 1.07E-02 | 3.61E-01 |
|  |  | 598 |  |  | MVMR- weighted median | -0.07 (-0.15, 0.01) | 0.93 (0.86, 1.01) | 8.99E-02 |  |
|  |  | 589 | 6.94 | 1.83E-27 | MVMR-IVW^b^ | -0.09 (-0.16, -0.02) | 0.91 (0.85, 0.98) | 9.47E-03 |  |
| Type 2 diabetes |  | 147 | 84.41 | 1.49E-23 | MVMR-IVW^a^ | 0.15 (0.10, 0.19) | 1.16 (1.11, 1.21) | **1.00E-10** |  |
|  |  | 147 |  |  | MVMR-Egger | 0.02 (-0.08, 0.11) | 1.02 (0.92, 1.12) | 7.26E-01 | 3.64E-03 |
|  |  | 147 |  |  | MVMR- weighted median | 0.13 (0.09, 0.18) | 1.14 (1.09, 1.19) | 1.68E-09 |  |
|  |  | 144 | 81.71 | 2.31E-23 | MVMR-IVW^b^ | 0.15 (0.10, 0.19) | 1.16 (1.11, 1.21) | 1.96E-10 |  |
| Two hours  glucose |  | 8 | 61.09 | 3.84E-06 | MVMR-IVW^a^ | 0.13 (-0.16, 0.43) | 1.14 (0.85, 1.54) | 3.74E-01 |  |
|  |  | 8 |  |  | MVMR-Egger | -0.42 (-1.39, 0.55) | 0.66 (0.25, 1.74) | 3.96E-01 | 2.41E-01 |
|  |  | 8 |  |  | MVMR- weighted median | 0.24 (0.01, 0.46) | 1.27 (1.01, 1.59) | 3.72E-02 |  |
|  |  | 7 | 57.26 | 1.58E-06 | MVMR-IVW^b^ | 0.13 (-0.19, 0.46) | 1.14 (0.83, 1.58) | 4.15E-01 |  |
| Total cholesterol |  | 284 | 284.39 | 3.88E-62 | MVMR-IVW^a^ | 0.47 (0.38, 0.56) | 1.60 (1.46, 1.75) | **1.68E-24** |  |
|  |  | 284 |  |  | MVMR-Egger | 0.66 (0.52, 0.81) | 1.94 (1.68, 2.24) | 2.80E-19 | 8.38E-04 |
|  |  | 284 |  |  | MVMR- weighted median | 0.44 (0.35, 0.53) | 1.55 (1.42, 1.70) | 9.24E-21 |  |
|  |  | 282 | 281.35 | 1.85E-56 | MVMR-IVW^b^ | 0.48 (0.39, 0.57) | 1.62 (1.48, 1.77) | 7.65E-27 |  |
| Triglycerides |  | 239 | 240.32 | 1.11E-50 | MVMR-IVW^a^ | 0.36 (0.26, 0.47) | 1.44 (1.30, 1.59) | **2.28E-12** |  |
|  |  | 239 |  |  | MVMR-Egger | 0.17 (0.01, 0.33) | 1.19 (1.01, 1.39) | 3.66E-02 | 2.44E-03 |
|  |  | 239 |  |  | MVMR- weighted median | 0.48 (0.36, 0.59) | 1.61 (1.43, 1.81) | 7.86E-16 |  |
|  |  | 238 | 239.86 | 1.75E-50 | MVMR-IVW^b^ | 0.37 (0.26, 0.47) | 1.44 (1.30, 1.60) | 1.82E-12 |  |
| Systolic blood  pressure |  | 400 | 77.08 | 4.08E-74 | MVMR-IVW^a^ | 0.03 (0.02, 0.04) | 1.03 (1.02, 1.04) | **9.48E-21** |  |
|  |  | 400 |  |  | MVMR-Egger | 0.03 (0.01, 0.04) | 1.03 (1.01, 1.04) | 4.40E-04 | 5.72E-01 |
|  |  | 400 |  |  | MVMR- weighted median | 0.03 (0.02, 0.04) | 1.03 (1.02, 1.04) | 3.66E-22 |  |
|  |  | 400 | 77.08 | 4.08E-74 | MVMR-IVW^b^ | 0.03 (0.02, 0.04) | 1.03 (1.02, 1.04) | 9.48E-21 |  |
| Omega 6 |  | 47 | 4.93 | 1.60E-52 | MVMR-IVW^a^ | 0.18 (-0.00, 0.36) | 1.19 (1.00, 1.43) | 5.00E-02 |  |
|  |  | 47 |  |  | MVMR-Egger | 0.20 (0.02, 0.38) | 1.22 (1.02, 1.46) | 3.01E-02 | 2.31E-01 |
|  |  | 47 |  |  | MVMR- weighted median | 0.20 (0.08, 0.32) | 1.23 (1.09, 1.38) | 8.56E-04 |  |
|  |  | 47 | 4.93 | 1.60E-52 | MVMR-IVW^b^ | 0.18 (-0.00, 0.36) | 1.19 (1.00, 1.43) | 5.00E-02 |  |
| Isoleucine |  | 7 | 104.58 | 2.10E-01 | MVMR-IVW^a^ | -0.23 (-0.47, 0.01) | 0.79 (0.62, 1.01) | 5.89E-02 |  |
|  |  | 7 |  |  | MVMR-Egger | -0.29 (-0.97, 0.39) | 0.75 (0.38, 1.48) | 4.04E-01 | 8.58E-01 |
|  |  | 7 |  |  | MVMR- weighted median | -0.22 (-0.51, 0.06) | 0.80 (0.60, 1.06) | 1.22E-01 |  |
|  |  | 7 | 104.58 | 2.10E-01 | MVMR-IVW^b^ | -0.23 (-0.47, 0.01) | 0.79 (0.62, 1.01) | 5.89E-02 |  |
| Leucine |  | 13 | 101.08 | 1.75E-01 | MVMR-IVW^a^ | -0.13 (-0.31, 0.06) | 0.88 (0.73, 1.06) | 1.79E-01 |  |
|  |  | 13 |  |  | MVMR-Egger | -0.25 (-0.66, 0.17) | 0.78 (0.51, 1.19) | 2.48E-01 | 5.29E-01 |
|  |  | 13 |  |  | MVMR- weighted median | -0.17 (-0.39, 0.06) | 0.85 (0.68, 1.06) | 1.49E-01 |  |
|  |  | 13 | 101.08 | 1.75E-01 | MVMR-IVW^b^ | -0.13 (-0.31, 0.06) | 0.88 (0.73, 1.06) | 1.79E-01 |  |
| Valine |  | 12 | 139.09 | 6.55E-02 | MVMR-IVW^a^ | -0.05 (-0.21, 0.11) | 0.95 (0.81, 1.12) | 5.47E-01 |  |
|  |  | 12 |  |  | MVMR-Egger | -0.12 (-0.43, 0.19) | 0.89 (0.65, 1.21) | 4.55E-01 | 6.04E-01 |
|  |  | 12 |  |  | MVMR- weighted median | -0.01 (-0.21, 0.19) | 0.99 (0.81, 1.21) | 9.36E-01 |  |
|  |  | 12 | 139.09 | 6.55E-02 | MVMR-IVW^b^ | -0.05 (-0.21, 0.11) | 0.95 (0.81, 1.12) | 5.47E-01 |  |
| Phenylalanine |  | 6 | 2.47 | 1.25E-01 | MVMR-IVW^a^ | -0.04 (-0.23, 0.15) | 0.96 (0.79, 1.16) | 6.51E-01 |  |
|  |  | 6 |  |  | MVMR-Egger | -0.06 (-0.31, 0.20) | 0.95 (0.73, 1.22) | 6.61E-01 | 8.46E-01 |
|  |  | 6 |  |  | MVMR- weighted median | 0.02 (-0.20, 0.24) | 1.02 (0.82, 1.27) | 8.50E-01 |  |
|  |  | 6 | 2.47 | 1.25E-01 | MVMR-IVW^b^ | -0.04 (-0.23, 0.15) | 0.96 (0.79, 1.16) | 6.51E-01 |  |
| Tyrosine |  | 18 | 146.49 | 3.61E-02 | MVMR-IVW^a^ | 0.07 (-0.08, 0.22) | 1.07 (0.92, 1.25) | 3.59E-01 |  |
|  |  | 18 |  |  | MVMR-Egger | 0.02 (-0.23, 0.28) | 1.02 (0.79, 1.32) | 8.60E-01 | 6.49E-01 |
|  |  | 18 |  |  | MVMR- weighted median | 0.18 (-0.04, 0.41) | 1.20 (0.96, 1.51) | 1.12E-01 |  |
|  |  | 18 | 146.49 | 3.61E-02 | MVMR-IVW^b^ | 0.07 (-0.08, 0.22) | 1.07 (0.92, 1.25) | 3.59E-01 |  |
| Alanine |  | 26 | 74.48 | 4.25E-01 | MVMR-IVW^a^ | 0.05 (-0.06, 0.16) | 1.05 (0.94, 1.17) | 3.78E-01 |  |
|  |  | 26 |  |  | MVMR-Egger | -0.03 (-0.34, 0.29) | 0.97 (0.71, 1.34) | 8.72E-01 | 6.16E-01 |
|  |  | 26 |  |  | MVMR- weighted median | 0.05 (-0.12, 0.22) | 1.05 (0.89, 1.24) | 5.77E-01 |  |
|  |  | 25 | 76.12 | 3.67E-01 | MVMR-IVW^b^ | 0.05 (-0.07, 0.17) | 1.05 (0.94, 1.18) | 4.03E-01 |  |
| Glycine |  | 37 | 197.30 | 2.22E-01 | MVMR-IVW^a^ | -0.09 (-0.16, -0.01) | 0.92 (0.85, 0.99) | 2.36E-02 |  |
|  |  | 37 |  |  | MVMR-Egger | -0.01 (-0.12, 0.10) | 0.99 (0.88, 1.10) | 8.23E-01 | 8.70E-02 |
|  |  | 37 |  |  | MVMR- weighted median | -0.17 (-0.28, -0.06) | 0.84 (0.75, 0.94) | 2.57E-03 |  |
|  |  | 37 | 197.30 | 2.22E-01 | MVMR-IVW^b^ | -0.09 (-0.16, -0.01) | 0.92 (0.85, 0.99) | 2.36E-02 |  |
| Body mass index | Myocardial  infarction | 449 | 73.06 | 3.87E-07 | MVMR-IVW^a^ | 0.50 (0.42, 0.58) | 1.65 (1.52, 1.79) | **1.85E-33** |  |
|  |  | 449 |  |  | MVMR-Egger | 0.57 (0.35, 0.79) | 1.77 (1.42, 2.21) | 4.68E-07 | 5.04E-01 |
|  |  | 449 |  |  | MVMR- weighted median | 0.56 (0.45, 0.67) | 1.75 (1.57, 1.95) | 3.17E-25 |  |
|  |  | 448 | 73.05 | 4.57E-07 | MVMR-IVW^b^ | 0.50 (0.42, 0.58) | 1.65 (1.52, 1.79) | 2.16E-33 |  |
| Height |  | 963 | 250.04 | 1.41E-22 | MVMR-IVW^a^ | -0.07 (-0.13, -0.01) | 0.93 (0.88, 0.99) | 1.49E-02 |  |
|  |  | 963 |  |  | MVMR-Egger | -0.09 (-0.20, 0.01) | 0.91 (0.82, 1.01) | 6.90E-02 | 5.98E-01 |
|  |  | 963 |  |  | MVMR- weighted median | -0.04 (-0.11, 0.03) | 0.96 (0.90, 1.03) | 2.92E-01 |  |
|  |  | 959 | 250.09 | 7.22E-23 | MVMR-IVW^b^ | -0.07 (-0.13, -0.01) | 0.93 (0.88, 0.99) | 1.67E-02 |  |
| Appendicular  lean mass |  | 595 | 7.71 | 5.38E-20 | MVMR-IVW^a^ | -0.05 (-0.13, 0.02) | 0.95 (0.88, 1.02) | 1.41E-01 |  |
|  |  | 595 |  |  | MVMR-Egger | -0.07 (-0.14, 0.01) | 0.94 (0.87, 1.01) | 8.61E-02 | 1.63E-01 |
|  |  | 595 |  |  | MVMR- weighted median | -0.08 (-0.17, 0.01) | 0.92 (0.85, 1.01) | 7.69E-02 |  |
|  |  | 586 | 6.97 | 2.79E-18 | MVMR-IVW^b^ | -0.06 (-0.13, 0.01) | 0.94 (0.87, 1.01) | 9.62E-02 |  |
| Type 2 diabetes |  | 148 | 84.09 | 1.56E-19 | MVMR-IVW^a^ | 0.14 (0.09, 0.19) | 1.15 (1.10, 1.21) | **4.23E-09** |  |
|  |  | 148 |  |  | MVMR-Egger | 0.00 (-0.10, 0.10) | 1.00 (0.90, 1.11) | 9.82E-01 | 2.68E-03 |
|  |  | 148 |  |  | MVMR- weighted median | 0.13 (0.08, 0.18) | 1.14 (1.08, 1.19) | 2.49E-07 |  |
|  |  | 145 | 81.41 | 1.21E-19 | MVMR-IVW^b^ | 0.14 (0.09, 0.19) | 1.15 (1.10, 1.21) | 8.60E-09 |  |
| Two hours glucose |  | 8 | 61.09 | 1.31E-03 | MVMR-IVW^a^ | 0.08 (-0.18, 0.33) | 1.08 (0.84, 1.39) | 5.45E-01 |  |
|  |  | 8 |  |  | MVMR-Egger | -0.39 (-1.23, 0.44) | 0.67 (0.29, 1.56) | 3.56E-01 | 2.47E-01 |
|  |  | 8 |  |  | MVMR- weighted median | 0.11 (-0.13, 0.35) | 1.11 (0.88, 1.41) | 3.79E-01 |  |
|  |  | 7 | 57.26 | 5.74E-04 | MVMR-IVW^b^ | 0.08 (-0.20, 0.35) | 1.08 (0.82, 1.42) | 5.81E-01 |  |
| Total cholesterol |  | 284 | 284.39 | 1.13E-49 | MVMR-IVW^a^ | 0.43 (0.33, 0.52) | 1.53 (1.39, 1.68) | **7.31E-19** |  |
|  |  | 284 |  |  | MVMR-Egger | 0.61 (0.46, 0.76) | 1.84 (1.58, 2.15) | 4.66E-15 | 2.88E-03 |
|  |  | 284 |  |  | MVMR- weighted median | 0.42 (0.32, 0.52) | 1.53 (1.38, 1.69) | 1.10E-16 |  |
|  |  | 282 | 284.35 | 2.87E-44 | MVMR-IVW^b^ | 0.44 (0.35, 0.53) | 1.56 (1.42, 1.71) | 5.74E-21 |  |
| Triglycerides |  | 239 | 240.32 | 2.43E-33 | MVMR-IVW^a^ | 0.35 (0.25, 0.45) | 1.42 (1.28, 1.58) | **1.95E-11** |  |
|  |  | 239 |  |  | MVMR-Egger | 0.17 (0.00, 0.33) | 1.18 (1.00, 1.39) | 4.47E-02 | 4.12E-03 |
|  |  | 239 |  |  | MVMR- weighted median | 0.43 (0.31, 0.55) | 1.53 (1.36, 1.73) | 1.56E-12 |  |
|  |  | 238 | 239.86 | 3.52E-33 | MVMR-IVW^b^ | 0.35 (0.25, 0.46) | 1.43 (1.29, 1.58) | 1.51E-11 |  |
| Systolic blood  pressure |  | 400 | 77.08 | 4.49E-45 | MVMR-IVW^a^ | 0.03 (0.02, 0.03) | 1.03 (1.02, 1.03) | **4.78E-16** |  |
|  |  | 400 |  |  | MVMR-Egger | 0.03 (0.01, 0.04) | 1.03 (1.01, 1.04) | 1.79E-04 | 7.82E-01 |
|  |  | 400 |  |  | MVMR- weighted median | 0.03 (0.02, 0.04) | 1.03 (1.02, 1.04) | 5.60E-17 |  |
|  |  | 400 | 77.08 | 4.49E-45 | MVMR-IVW^b^ | 0.03 (0.02, 0.03) | 1.03 (1.02, 1.03) | 4.78E-16 |  |
| Omega 6 |  | 47 | 4.93 | 6.84E-39 | MVMR-IVW^a^ | 0.15 (-0.02, 0.33) | 1.17 (0.98, 1.39) | 8.42E-02 |  |
|  |  | 47 |  |  | MVMR-Egger | 0.18 (0.01, 0.36) | 1.20 (1.01, 1.43) | 4.32E-02 | 1.27E-01 |
|  |  | 47 |  |  | MVMR- weighted median | 0.16 (0.03, 0.29) | 1.17 (1.03, 1.34) | 1.95E-02 |  |
|  |  | 47 | 4.93 | 6.84E-39 | MVMR-IVW^b^ | 0.15 (-0.02, 0.33) | 1.17 (0.98, 1.39) | 8.42E-02 |  |
| Isoleucine |  | 7 | 104.58 | 9.07E-01 | MVMR-IVW^a^ | -0.29 (-0.51, -0.07) | 0.75 (0.60, 0.93) | 8.64E-03 |  |
|  |  | 7 |  |  | MVMR-Egger | -0.49 (-1.04, 0.06) | 0.61 (0.35, 1.06) | 7.94E-02 | 4.40E-01 |
|  |  | 7 |  |  | MVMR- weighted median | -0.32 (-0.64, -0.00) | 0.73 (0.53, 1.00) | 4.97E-02 |  |
|  |  | 7 | 104.58 | 9.07E-01 | MVMR-IVW^b^ | -0.29 (-0.51, -0.07) | 0.75 (0.60, 0.93) | 8.64E-03 |  |
| Leucine |  | 13 | 101.08 | 7.12E-01 | MVMR-IVW^a^ | -0.11 (-0.27, 0.05) | 0.90 (0.77, 1.05) | 1.74E-01 |  |
|  |  | 13 |  |  | MVMR-Egger | -0.33 (-0.67, 0.02) | 0.72 (0.51, 1.02) | 6.59E-02 | 1.70E-01 |
|  |  | 13 |  |  | MVMR- weighted median | -0.23 (-0.49, 0.03) | 0.79 (0.61, 1.03) | 8.36E-02 |  |
|  |  | 13 | 101.08 | 7.12E-01 | MVMR-IVW^b^ | -0.11 (-0.27, 0.05) | 0.90 (0.77, 1.05) | 1.74E-01 |  |
| Valine |  | 12 | 139.09 | 3.28E-01 | MVMR-IVW^a^ | -0.07 (-0.21, 0.07) | 0.93 (0.81, 1.08) | 3.40E-01 |  |
|  |  | 12 |  |  | MVMR-Egger | -0.16 (-0.44, 0.12) | 0.85 (0.64, 1.12) | 2.51E-01 | 4.41E-01 |
|  |  | 12 |  |  | MVMR- weighted median | -0.09 (-0.31, 0.13) | 0.91 (0.73, 1.13) | 4.01E-01 |  |
|  |  | 12 | 139.09 | 3.28E-01 | MVMR-IVW^b^ | -0.07 (-0.21, 0.07) | 0.93 (0.81, 1.08) | 3.40E-01 |  |
| Phenylalanine |  | 6 | 2.47 | 4.79E-03 | MVMR-IVW^a^ | -0.01 (-0.30, 0.28) | 0.99 (0.74, 1.32) | 9.57E-01 |  |
|  |  | 6 |  |  | MVMR-Egger | -0.02 (-0.41, 0.36) | 0.98 (0.66, 1.44) | 9.08E-01 | 8.82E-01 |
|  |  | 6 |  |  | MVMR- weighted median | 0.10 (-0.20, 0.40) | 1.11 (0.82, 1.49) | 5.11E-01 |  |
|  |  | 6 | 2.47 | 4.79E-03 | MVMR-IVW^b^ | -0.01 (-0.30, 0.28) | 0.99 (0.74, 1.32) | 9.57E-01 |  |
| Tyrosine |  | 18 | 146.49 | 3.00E-02 | MVMR-IVW^a^ | 0.06 (-0.10, 0.22) | 1.06 (0.90, 1.25) | 4.79E-01 |  |
|  |  | 18 |  |  | MVMR-Egger | 0.00 (-0.28, 0.28) | 1.00 (0.76, 1.32) | 9.98E-01 | 6.05E-01 |
|  |  | 18 |  |  | MVMR- weighted median | 0.10 (-0.14, 0.33) | 1.10 (0.87, 1.39) | 4.16E-01 |  |
|  |  | 18 | 146.49 | 3.00E-02 | MVMR-IVW^b^ | 0.06 (-0.10, 0.22) | 1.06 (0.90, 1.25) | 4.79E-01 |  |
| Alanine |  | 26 | 74.48 | 4.80E-01 | MVMR-IVW^a^ | 0.01 (-0.12, 0.13) | 1.01 (0.89, 1.14) | 9.05E-01 |  |
|  |  | 26 |  |  | MVMR-Egger | -0.11 (-0.45, 0.23) | 0.90 (0.64, 1.26) | 5.27E-01 | 4.70E-01 |
|  |  | 26 |  |  | MVMR- weighted median | -0.04 (-0.22, 0.14) | 0.96 (0.80, 1.15) | 6.32E-01 |  |
|  |  | 25 | 76.12 | 4.26E-01 | MVMR-IVW^b^ | 0.01 (-0.11, 0.14) | 1.01 (0.89, 1.15) | 8.52E-01 |  |
| Glycine |  | 35 | 198.79 | 9.56E-02 | MVMR-IVW^a^ | -0.02 (-0.11, 0.07) | 0.98 (0.90, 1.07) | 7.06E-01 |  |
|  |  | 35 |  |  | MVMR-Egger | 0.04 (-0.10, 0.18) | 1.04 (0.91, 1.19) | 5.61E-01 | 2.80E-01 |
|  |  | 35 |  |  | MVMR- weighted median | -0.01 (-0.13, 0.11) | 0.99 (0.88, 1.12) | 8.57E-01 |  |
|  |  | 35 | 198.79 | 9.56E-02 | MVMR-IVW^b^ | -0.02 (-0.11, 0.07) | 0.98 (0.90, 1.07) | 7.06E-01 |  |
| Body mass index | Angina  pectoris | 440 | 73.16 | 4.12E-20 | MVMR-IVW^a^ | 0.30 (0.23, 0.38) | 1.35 (1.26, 1.46) | **2.09E-15** |  |
|  |  | 440 |  |  | MVMR-Egger | 0.32 (0.11, 0.52) | 1.37 (1.12, 1.68) | 2.74E-03 | 9.03E-01 |
|  |  | 440 |  |  | MVMR- weighted median | 0.36 (0.28, 0.45) | 1.44 (1.32, 1.57) | 1.35E-16 |  |
|  |  | 439 | 73.15 | 3.02E-20 | MVMR-IVW^b^ | 0.30 (0.23, 0.38) | 1.35 (1.26, 1.46) | 2.14E-15 |  |
| Height |  | 962 | 250.36 | 1.32E-64 | MVMR-IVW^a^ | -0.06 (-0.11, -0.01) | 0.94 (0.89, 0.99) | 3.10E-02 |  |
|  |  | 962 |  |  | MVMR-Egger | -0.00 (-0.10, 0.09) | 1.00 (0.91, 1.09) | 9.23E-01 | 1.64E-01 |
|  |  | 962 |  |  | MVMR- weighted median | -0.07 (-0.13, -0.01) | 0.93 (0.88, 0.99) | 2.23E-02 |  |
|  |  | 958 | 250.42 | 3.63E-65 | MVMR-IVW^b^ | -0.06 (-0.12, -0.01) | 0.94 (0.89, 0.99) | 2.64E-02 |  |
| Appendicular lean mass |  | 585 | 7.88 | 3.13E-57 | MVMR-IVW^a^ | -0.07 (-0.14, 0.00) | 0.93 (0.87, 1.00) | 5.03E-02 |  |
|  |  | 585 |  |  | MVMR-Egger | -0.06 (-0.13, 0.01) | 0.94 (0.88, 1.01) | 8.27E-02 | 3.76E-01 |
|  |  | 585 |  |  | MVMR- weighted median | -0.06 (-0.14, 0.01) | 0.94 (0.87, 1.01) | 1.11E-01 |  |
|  |  | 576 | 7.12 | 1.80E-53 | MVMR-IVW^b^ | -0.07 (-0.14, -0.01) | 0.93 (0.87, 0.99) | 3.19E-02 |  |
| Type 2 diabetes |  | 144 | 84.61 | 1.05E-52 | MVMR-IVW^a^ | 0.12 (0.07, 0.17) | 1.13 (1.08, 1.18) | **9.47E-07** |  |
|  |  | 144 |  |  | MVMR-Egger | 0.08 (-0.02, 0.19) | 1.09 (0.98, 1.20) | 1.20E-01 | 4.16E-01 |
|  |  | 144 |  |  | MVMR- weighted median | 0.12 (0.08, 0.16) | 1.13 (1.08, 1.17) | 4.38E-09 |  |
|  |  | 141 | 81.86 | 2.34E-52 | MVMR-IVW^b^ | 0.12 (0.07, 0.17) | 1.13 (1.07, 1.18) | 4.09E-06 |  |
| Two hours glucose |  | 8 | 61.09 | 1.49E-04 | MVMR-IVW^a^ | 0.15 (-0.07, 0.38) | 1.16 (0.93, 1.46) | 1.86E-01 |  |
|  |  | 8 |  |  | MVMR-Egger | -0.27 (-0.99, 0.45) | 0.77 (0.37, 1.58) | 4.69E-01 | 2.32E-01 |
|  |  | 8 |  |  | MVMR- weighted median | 0.29 (0.04, 0.53) | 1.33 (1.04, 1.69) | 2.07E-02 |  |
|  |  | 7 | 57.26 | 5.48E-05 | MVMR-IVW^b^ | 0.15 (-0.10, 0.40) | 1.17 (0.91, 1.49) | 2.26E-01 |  |
| Total cholesterol |  | 288 | 290.96 | 4.41E-104 | MVMR-IVW^a^ | 0.34 (0.25, 0.42) | 1.40 (1.29, 1.53) | **4.74E-15** |  |
|  |  | 288 |  |  | MVMR-Egger | 0.52 (0.39, 0.65) | 1.69 (1.48, 1.92) | 1.44E-14 | 5.48E-04 |
|  |  | 288 |  |  | MVMR- weighted median | 0.23 (0.15, 0.32) | 1.26 (1.16, 1.38) | 1.75E-07 |  |
|  |  | 286 | 290.96 | 5.68E-100 | MVMR-IVW^b^ | 0.35 (0.27, 0.44) | 1.42 (1.31, 1.55) | 2.32E-16 |  |
| Triglycerides |  | 246 | 246.24 | 3.07E-51 | MVMR-IVW^a^ | 0.41 (0.33, 0.49) | 1.51 (1.39, 1.63) | **2.58E-23** |  |
|  |  | 246 |  |  | MVMR-Egger | 0.31 (0.19, 0.43) | 1.36 (1.21, 1.54) | 6.97E-07 | 3.85E-02 |
|  |  | 246 |  |  | MVMR- weighted median | 0.45 (0.37, 0.53) | 1.57 (1.45, 1.69) | 1.23E-29 |  |
|  |  | 245 | 245.82 | 1.90E-51 | MVMR-IVW^b^ | 0.41 (0.33, 0.49) | 1.51 (1.39, 1.63) | 3.80E-23 |  |
| Systolic blood pressure |  | 396 | 77.75 | 3.01E-102 | MVMR-IVW^a^ | 0.03 (0.03, 0.04) | 1.03 (1.03, 1.04) | **5.92E-27** |  |
|  |  | 396 |  |  | MVMR-Egger | 0.04 (0.03, 0.05) | 1.04 (1.03, 1.06) | 5.50E-08 | 3.45E-01 |
|  |  | 396 |  |  | MVMR- weighted median | 0.04 (0.03, 0.04) | 1.04 (1.03, 1.04) | 1.47E-34 |  |
|  |  | 396 | 77.75 | 3.01E-102 | MVMR-IVW^b^ | 0.03 (0.03, 0.04) | 1.03 (1.03, 1.04) | 5.92E-27 |  |
| Omega 6 |  | 46 | 4.84 | 1.94E-58 | MVMR-IVW^a^ | 0.19 (0.02, 0.36) | 1.21 (1.02, 1.44) | 3.00E-02 |  |
|  |  | 46 |  |  | MVMR-Egger | 0.20 (0.02, 0.37) | 1.22 (1.02, 1.45) | 2.55E-02 | 4.68E-01 |
|  |  | 46 |  |  | MVMR- weighted median | 0.19 (0.06, 0.32) | 1.21 (1.06, 1.38) | 4.07E-03 |  |
|  |  | 46 | 4.84 | 1.94E-58 | MVMR-IVW^b^ | 0.19 (0.02, 0.36) | 1.21 (1.02, 1.44) | 3.00E-02 |  |
| Isoleucine |  | 7 | 104.58 | 1.30E-06 | MVMR-IVW^a^ | 0.06 (-0.40, 0.52) | 1.06 (0.67, 1.68) | 7.90E-01 |  |
|  |  | 7 |  |  | MVMR-Egger | -0.10 (-1.40, 1.21) | 0.91 (0.25, 3.34) | 8.86E-01 | 7.97E-01 |
|  |  | 7 |  |  | MVMR- weighted median | 0.11 (-0.27, 0.48) | 1.11 (0.76, 1.62) | 5.84E-01 |  |
|  |  | 7 | 104.58 | 1.30E-06 | MVMR-IVW^b^ | 0.06 (-0.40, 0.52) | 1.06 (0.67, 1.68) | 7.90E-01 |  |
| Leucine |  | 12 | 106.74 | 1.86E-04 | MVMR-IVW^a^ | 0.12 (-0.12, 0.36) | 1.13 (0.89, 1.43) | 3.27E-01 |  |
|  |  | 12 |  |  | MVMR-Egger | -0.18 (-0.69, 0.34) | 0.84 (0.50, 1.40) | 4.95E-01 | 2.04E-01 |
|  |  | 12 |  |  | MVMR- weighted median | 0.15 (-0.13, 0.44) | 1.17 (0.88, 1.56) | 2.91E-01 |  |
|  |  | 12 | 106.74 | 1.86E-04 | MVMR-IVW^b^ | 0.12 (-0.12, 0.36) | 1.13 (0.89, 1.43) | 3.27E-01 |  |
| Valine |  | 12 | 148.51 | 5.15E-05 | MVMR-IVW^a^ | 0.19 (-0.01, 0.39) | 1.21 (0.99, 1.47) | 5.90E-02 |  |
|  |  | 12 |  |  | MVMR-Egger | -0.10 (-0.41, 0.22) | 0.91 (0.66, 1.25) | 5.51E-01 | 3.54E-02 |
|  |  | 12 |  |  | MVMR- weighted median | 0.22 (-0.01, 0.45) | 1.24 (0.99, 1.56) | 5.79E-02 |  |
|  |  | 12 | 148.51 | 5.15E-05 | MVMR-IVW^b^ | 0.19 (-0.01, 0.39) | 1.21 (0.99, 1.47) | 5.90E-02 |  |
| Phenylalanine |  | 5 | 2.58 | 1.58E-03 | MVMR-IVW^a^ | 0.03 (-0.25, 0.31) | 1.03 (0.78, 1.37) | 8.40E-01 |  |
|  |  | 5 |  |  | MVMR-Egger | 0.17 (-0.10, 0.44) | 1.18 (0.90, 1.55) | 2.20E-01 | 8.25E-02 |
|  |  | 5 |  |  | MVMR- weighted median | 0.08 (-0.23, 0.39) | 1.08 (0.80, 1.47) | 6.12E-01 |  |
|  |  | 5 | 2.58 | 1.58E-03 | MVMR-IVW^b^ | 0.03 (-0.25, 0.31) | 1.03 (0.78, 1.37) | 8.40E-01 |  |
| Tyrosine |  | 18 | 146.49 | 1.33E-04 | MVMR-IVW^a^ | 0.19 (0.05, 0.33) | 1.21 (1.05, 1.39) | 8.13E-03 |  |
|  |  | 18 |  |  | MVMR-Egger | 0.14 (-0.09, 0.36) | 1.15 (0.92, 1.44) | 2.27E-01 | 5.74E-01 |
|  |  | 18 |  |  | MVMR- weighted median | 0.22 (0.08, 0.37) | 1.25 (1.08, 1.44) | 2.24E-03 |  |
|  |  | 18 | 146.49 | 1.33E-04 | MVMR-IVW^b^ | 0.19 (0.05, 0.33) | 1.21 (1.05, 1.39) | 8.13E-03 |  |
| Alanine |  | 27 | 72.89 | 2.42E-03 | MVMR-IVW^a^ | 0.26 (0.13, 0.40) | 1.30 (1.14, 1.49) | **1.23E-04** |  |
|  |  | 27 |  |  | MVMR-Egger | 0.22 (-0.18, 0.62) | 1.25 (0.84, 1.86) | 2.77E-01 | 8.25E-01 |
|  |  | 27 |  |  | MVMR- weighted median | 0.30 (0.14, 0.46) | 1.35 (1.15, 1.58) | 2.20E-04 |  |
|  |  | 26 | 74.40 | 1.62E-03 | MVMR-IVW^b^ | 0.26 (0.12, 0.40) | 1.30 (1.13, 1.50) | 2.44E-04 |  |
| Glycine |  | 37 | 203.11 | 3.87E-02 | MVMR-IVW^a^ | -0.05 (-0.13, 0.02) | 0.95 (0.88, 1.02) | 1.72E-01 |  |
|  |  | 37 |  |  | MVMR-Egger | -0.03 (-0.14, 0.08) | 0.97 (0.87, 1.08) | 5.73E-01 | 5.96E-01 |
|  |  | 37 |  |  | MVMR- weighted median | -0.07 (-0.17, 0.03) | 0.93 (0.84, 1.03) | 1.90E-01 |  |
|  |  | 37 | 203.11 | 3.87E-02 | MVMR-IVW^b^ | -0.05 (-0.13, 0.02) | 0.95 (0.88, 1.02) | 1.72E-01 |  |
| Body mass index | Aortic  aneurysm | 440 | 73.16 | 2.85E-07 | MVMR-IVW^a^ | 0.40 (0.28, 0.53) | 1.50 (1.32, 1.69) | **1.47E-10** |  |
|  |  | 440 |  |  | MVMR-Egger | 0.42 (0.09, 0.76) | 1.53 (1.09, 2.15) | 1.41E-02 | 8.91E-01 |
|  |  | 440 |  |  | MVMR- weighted median | 0.42 (0.26, 0.59) | 1.53 (1.30, 1.80) | 2.36E-07 |  |
|  |  | 439 | 73.15 | 6.19E-07 | MVMR-IVW^b^ | 0.40 (0.28, 0.52) | 1.49 (1.32, 1.69) | 1.50E-10 |  |
| Height |  | 962 | 250.36 | 8.08E-22 | MVMR-IVW^a^ | 0.13 (0.05, 0.22) | 1.14 (1.05, 1.25) | 2.26E-03 |  |
|  |  | 962 |  |  | MVMR-Egger | 0.24 (0.09, 0.40) | 1.28 (1.10, 1.48) | 1.51E-03 | 8.29E-02 |
|  |  | 962 |  |  | MVMR- weighted median | 0.13 (0.02, 0.24) | 1.14 (1.02, 1.27) | 1.60E-02 |  |
|  |  | 958 | 250.42 | 4.89E-22 | MVMR-IVW^b^ | 0.13 (0.05, 0.22) | 1.14 (1.05, 1.25) | 2.73E-03 |  |
| Appendicular lean mass |  | 585 | 7.88 | 1.03E-18 | MVMR-IVW^a^ | 0.22 (0.11, 0.32) | 1.24 (1.11, 1.38) | **9.17E-05** |  |
|  |  | 585 |  |  | MVMR-Egger | 0.23 (0.12, 0.34) | 1.26 (1.12, 1.40) | 4.99E-05 | 2.68E-01 |
|  |  | 585 |  |  | MVMR- weighted median | 0.27 (0.14, 0.40) | 1.31 (1.15, 1.49) | 5.63E-05 |  |
|  |  | 576 | 7.12 | 3.69E-17 | MVMR-IVW^b^ | 0.20 (0.09, 0.31) | 1.22 (1.10, 1.36) | 2.41E-04 |  |
| Type 2 diabetes |  | 144 | 84.61 | 3.69E-05 | MVMR-IVW^a^ | -0.07 (-0.12, -0.01) | 0.93 (0.88, 0.99) | 1.62E-02 |  |
|  |  | 144 |  |  | MVMR-Egger | -0.14 (-0.26, -0.02) | 0.87 (0.77, 0.98) | 2.13E-02 | 1.84E-01 |
|  |  | 144 |  |  | MVMR- weighted median | -0.08 (-0.15, -0.01) | 0.92 (0.86, 0.99) | 2.48E-02 |  |
|  |  | 141 | 81.86 | 4.49E-05 | MVMR-IVW^b^ | -0.06 (-0.11, 0.00) | 0.95 (0.89, 1.00) | 5.48E-02 |  |
| Two hours glucose |  | 8 | 61.09 | 1.10E-01 | MVMR-IVW^a^ | -0.36 (-0.62, -0.10) | 0.70 (0.54, 0.91) | 6.91E-03 |  |
|  |  | 8 |  |  | MVMR-Egger | -1.27 (-1.95, -0.59) | 0.28 (0.14, 0.55) | 2.46E-04 | 5.65E-03 |
|  |  | 8 |  |  | MVMR- weighted median | -0.37 (-0.71, -0.04) | 0.69 (0.49, 0.96) | 2.94E-02 |  |
|  |  | 7 | 57.26 | 1.62E-01 | MVMR-IVW^b^ | -0.37 (-0.62, -0.12) | 0.69 (0.54, 0.89) | 4.29E-03 |  |
| Total cholesterol |  | 288 | 290.96 | 1.35E-17 | MVMR-IVW^a^ | 0.15 (0.04, 0.25) | 1.16 (1.04, 1.29) | 7.15E-03 |  |
|  |  | 288 |  |  | MVMR-Egger | 0.17 (-0.00, 0.34) | 1.19 (1.00, 1.41) | 5.13E-02 | 7.32E-01 |
|  |  | 288 |  |  | MVMR- weighted median | 0.17 (0.04, 0.31) | 1.19 (1.04, 1.36) | 1.16E-02 |  |
|  |  | 286 | 290.96 | 3.80E-15 | MVMR-IVW^b^ | 0.17 (0.06, 0.27) | 1.18 (1.06, 1.31) | 1.90E-03 |  |
| Triglycerides |  | 246 | 246.24 | 5.69E-21 | MVMR-IVW^a^ | 0.30 (0.17, 0.42) | 1.35 (1.19, 1.53) | **2.98E-06** |  |
|  |  | 246 |  |  | MVMR-Egger | 0.29 (0.09, 0.48) | 1.33 (1.10, 1.61) | 3.59E-03 | 8.60E-01 |
|  |  | 246 |  |  | MVMR- weighted median | 0.29 (0.16, 0.43) | 1.34 (1.17, 1.53) | 2.78E-05 |  |
|  |  | 245 | 245.82 | 4.29E-20 | MVMR-IVW^b^ | 0.31 (0.19, 0.44) | 1.37 (1.21, 1.55) | 9.97E-07 |  |
| Systolic blood pressure |  | 395 | 77.85 | 1.74E-57 | MVMR-IVW^a^ | 0.00 (-0.01, 0.01) | 1.00 (0.99, 1.01) | 3.87E-01 |  |
|  |  | 395 |  |  | MVMR-Egger | 0.03 (0.00, 0.05) | 1.03 (1.00, 1.05) | 3.17E-02 | 4.92E-02 |
|  |  | 395 |  |  | MVMR- weighted median | 0.01 (0.00, 0.02) | 1.01 (1.00, 1.02) | 7.71E-03 |  |
|  |  | 395 | 77.85 | 1.74E-57 | MVMR-IVW^b^ | 0.00 (-0.01, 0.01) | 1.00 (0.99, 1.01) | 3.87E-01 |  |
| Omega 6 |  | 46 | 4.84 | 1.24E-16 | MVMR-IVW^a^ | 0.12 (-0.08, 0.32) | 1.12 (0.92, 1.37) | 2.47E-01 |  |
|  |  | 46 |  |  | MVMR-Egger | 0.13 (-0.07, 0.33) | 1.14 (0.94, 1.39) | 1.93E-01 | 2.50E-01 |
|  |  | 46 |  |  | MVMR- weighted median | -0.04 (-0.25, 0.17) | 0.96 (0.78, 1.19) | 7.23E-01 |  |
|  |  | 46 | 4.84 | 1.24E-16 | MVMR-IVW^b^ | 0.12 (-0.08, 0.32) | 1.12 (0.92, 1.37) | 2.47E-01 |  |
| Isoleucine |  | 7 | 104.58 | 6.75E-01 | MVMR-IVW^a^ | -0.41 (-0.73, -0.09) | 0.66 (0.48, 0.92) | 1.24E-02 |  |
|  |  | 7 |  |  | MVMR-Egger | -0.08 (-0.90, 0.74) | 0.93 (0.41, 2.11) | 8.55E-01 | 3.88E-01 |
|  |  | 7 |  |  | MVMR- weighted median | -0.58 (-1.04, -0.12) | 0.56 (0.35, 0.89) | 1.38E-02 |  |
|  |  | 7 | 104.58 | 6.75E-01 | MVMR-IVW^b^ | -0.41 (-0.73, -0.09) | 0.66 (0.48, 0.92) | 1.24E-02 |  |
| Leucine |  | 12 | 106.74 | 2.09E-02 | MVMR-IVW^a^ | -0.08 (-0.40, 0.24) | 0.92 (0.67, 1.27) | 6.08E-01 |  |
|  |  | 12 |  |  | MVMR-Egger | -0.20 (-0.95, 0.55) | 0.82 (0.39, 1.73) | 6.02E-01 | 7.36E-01 |
|  |  | 12 |  |  | MVMR- weighted median | -0.23 (-0.58, 0.12) | 0.79 (0.56, 1.13) | 1.96E-01 |  |
|  |  | 12 | 106.74 | 2.09E-02 | MVMR-IVW^b^ | -0.08 (-0.40, 0.24) | 0.92 (0.67, 1.27) | 6.08E-01 |  |
| Valine |  | 12 | 148.51 | 8.02E-02 | MVMR-IVW^a^ | -0.09 (-0.33, 0.15) | 0.91 (0.72, 1.16) | 4.43E-01 |  |
|  |  | 12 |  |  | MVMR-Egger | 0.09 (-0.36, 0.53) | 1.09 (0.70, 1.71) | 7.07E-01 | 3.51E-01 |
|  |  | 12 |  |  | MVMR- weighted median | -0.08 (-0.41, 0.24) | 0.92 (0.66, 1.28) | 6.18E-01 |  |
|  |  | 12 | 148.51 | 8.02E-02 | MVMR-IVW^b^ | -0.09 (-0.33, 0.15) | 0.91 (0.72, 1.16) | 4.43E-01 |  |
| Phenylalanine |  | 5 | 2.58 | 1.50E-01 | MVMR-IVW^a^ | -0.03 (-0.32, 0.25) | 0.97 (0.72, 1.29) | 8.15E-01 |  |
|  |  | 5 |  |  | MVMR-Egger | -0.17 (-0.47, 0.12) | 0.84 (0.63, 1.13) | 2.56E-01 | 1.20E-01 |
|  |  | 5 |  |  | MVMR- weighted median | -0.07 (-0.41, 0.26) | 0.93 (0.66, 1.30) | 6.75E-01 |  |
|  |  | 5 | 2.58 | 1.50E-01 | MVMR-IVW^b^ | -0.03 (-0.32, 0.25) | 0.97 (0.72, 1.29) | 8.15E-01 |  |
| Tyrosine |  | 18 | 146.49 | 1.52E-06 | MVMR-IVW^a^ | 0.13 (-0.14, 0.40) | 1.14 (0.87, 1.49) | 3.38E-01 |  |
|  |  | 18 |  |  | MVMR-Egger | -0.07 (-0.49, 0.34) | 0.93 (0.61, 1.40) | 7.26E-01 | 2.08E-01 |
|  |  | 18 |  |  | MVMR- weighted median | -0.02 (-0.28, 0.24) | 0.98 (0.76, 1.28) | 8.89E-01 |  |
|  |  | 18 | 146.49 | 1.52E-06 | MVMR-IVW^b^ | 0.13 (-0.14, 0.40) | 1.14 (0.87, 1.49) | 3.38E-01 |  |
| Alanine |  | 27 | 72.89 | 2.30E-01 | MVMR-IVW^a^ | -0.02 (-0.22, 0.17) | 0.98 (0.80, 1.19) | 8.11E-01 |  |
|  |  | 27 |  |  | MVMR-Egger | 0.05 (-0.53, 0.62) | 1.05 (0.59, 1.86) | 8.75E-01 | 8.00E-01 |
|  |  | 27 |  |  | MVMR- weighted median | 0.03 (-0.23, 0.30) | 1.03 (0.79, 1.35) | 8.06E-01 |  |
|  |  | 26 | 74.40 | 3.34E-01 | MVMR-IVW^b^ | 0.01 (-0.18, 0.20) | 1.01 (0.84, 1.22) | 9.25E-01 |  |
| Glycine |  | 37 | 203.11 | 1.63E-02 | MVMR-IVW^a^ | 0.12 (-0.01, 0.25) | 1.13 (0.99, 1.28) | 6.49E-02 |  |
|  |  | 37 |  |  | MVMR-Egger | 0.07 (-0.11, 0.26) | 1.08 (0.90, 1.29) | 4.26E-01 | 4.94E-01 |
|  |  | 37 |  |  | MVMR- weighted median | 0.19 (0.02, 0.36) | 1.21 (1.02, 1.43) | 2.87E-02 |  |
|  |  | 37 | 203.11 | 1.63E-02 | MVMR-IVW^b^ | 0.12 (-0.01, 0.25) | 1.13 (0.99, 1.28) | 6.49E-02 |  |
| Body mass index | Venous  thromboembolism | 440 | 73.16 | 1.42E-10 | MVMR-IVW^a^ | 0.36 (0.28, 0.44) | 1.43 (1.32, 1.55) | **9.93E-19** |  |
|  |  | 440 |  |  | MVMR-Egger | 0.58 (0.36, 0.80) | 1.78 (1.44, 2.22) | 1.75E-07 | 3.21E-02 |
|  |  | 440 |  |  | MVMR- weighted median | 0.31 (0.21, 0.41) | 1.36 (1.24, 1.50) | 4.91E-10 |  |
|  |  | 439 | 73.15 | 1.15E-10 | MVMR-IVW^b^ | 0.36 (0.28, 0.44) | 1.43 (1.32, 1.55) | 1.11E-18 |  |
| Height |  | 962 | 250.36 | 1.91E-22 | MVMR-IVW^a^ | 0.22 (0.16, 0.27) | 1.24 (1.18, 1.31) | **2.21E-15** |  |
|  |  | 962 |  |  | MVMR-Egger | 0.26 (0.16, 0.35) | 1.29 (1.18, 1.42) | 9.00E-08 | 3.28E-01 |
|  |  | 962 |  |  | MVMR- weighted median | 0.19 (0.12, 0.25) | 1.20 (1.13, 1.29) | 3.72E-08 |  |
|  |  | 958 | 250.42 | 2.81E-22 | MVMR-IVW^b^ | 0.22 (0.17, 0.27) | 1.25 (1.18, 1.31) | 2.12E-15 |  |
| Appendicular lean mass |  | 585 | 7.88 | 9.26E-39 | MVMR-IVW^a^ | 0.18 (0.11, 0.25) | 1.20 (1.11, 1.29) | **1.70E-06** |  |
|  |  | 585 |  |  | MVMR-Egger | 0.18 (0.11, 0.26) | 1.20 (1.11, 1.29) | 2.01E-06 | 7.30E-01 |
|  |  | 585 |  |  | MVMR- weighted median | 0.19 (0.11, 0.28) | 1.21 (1.11, 1.32) | 8.65E-06 |  |
|  |  | 576 | 7.12 | 2.60E-35 | MVMR-IVW^b^ | 0.17 (0.10, 0.25) | 1.19 (1.11, 1.28) | 3.38E-06 |  |
| Type 2 diabetes |  | 144 | 84.61 | 2.04E-10 | MVMR-IVW^a^ | -0.05 (-0.08, -0.01) | 0.96 (0.92, 0.99) | 2.08E-02 |  |
|  |  | 144 |  |  | MVMR-Egger | -0.13 (-0.21, -0.05) | 0.88 (0.81, 0.95) | 1.99E-03 | 2.40E-02 |
|  |  | 144 |  |  | MVMR- weighted median | -0.03 (-0.07, 0.02) | 0.97 (0.93, 1.02) | 1.97E-01 |  |
|  |  | 141 | 81.86 | 3.06E-10 | MVMR-IVW^b^ | -0.04 (-0.08, 0.00) | 0.96 (0.93, 1.00) | 6.53E-02 |  |
| Two hours glucose |  | 8 | 61.09 | 2.39E-03 | MVMR-IVW^a^ | -0.31 (-0.55, -0.08) | 0.73 (0.58, 0.93) | 9.10E-03 |  |
|  |  | 8 |  |  | MVMR-Egger | -0.67 (-1.45, 0.11) | 0.51 (0.23, 1.12) | 9.31E-02 | 3.46E-01 |
|  |  | 8 |  |  | MVMR- weighted median | -0.19 (-0.41, 0.02) | 0.82 (0.66, 1.02) | 7.40E-02 |  |
|  |  | 7 | 57.26 | 2.16E-03 | MVMR-IVW^b^ | -0.32 (-0.57, -0.07) | 0.73 (0.57, 0.94) | 1.31E-02 |  |
| Total cholesterol |  | 288 | 290.96 | 3.21E-41 | MVMR-IVW^a^ | -0.09 (-0.17, -0.01) | 0.91 (0.84, 0.99) | 2.12E-02 |  |
|  |  | 288 |  |  | MVMR-Egger | -0.08 (-0.21, 0.04) | 0.92 (0.81, 1.04) | 1.83E-01 | 8.86E-01 |
|  |  | 288 |  |  | MVMR- weighted median | -0.08 (-0.17, 0.01) | 0.92 (0.84, 1.01) | 8.22E-02 |  |
|  |  | 286 | 290.96 | 6.58E-38 | MVMR-IVW^b^ | -0.08 (-0.16, -0.00) | 0.92 (0.86, 1.00) | 4.45E-02 |  |
| Triglycerides |  | 246 | 246.24 | 3.86E-28 | MVMR-IVW^a^ | -0.13 (-0.21, -0.04) | 0.88 (0.81, 0.96) | 2.60E-03 |  |
|  |  | 246 |  |  | MVMR-Egger | -0.16 (-0.29, -0.04) | 0.85 (0.75, 0.97) | 1.22E-02 | 4.74E-01 |
|  |  | 246 |  |  | MVMR- weighted median | -0.08 (-0.18, 0.01) | 0.92 (0.84, 1.01) | 8.00E-02 |  |
|  |  | 245 | 245.82 | 3.07E-28 | MVMR-IVW^b^ | -0.12 (-0.21, -0.04) | 0.88 (0.81, 0.96) | 3.45E-03 |  |
| Systolic blood pressure |  | 395 | 77.85 | 1.82E-17 | MVMR-IVW^a^ | -0.01 (-0.02, -0.01) | 0.99 (0.98, 0.99) | **4.62E-06** |  |
|  |  | 395 |  |  | MVMR-Egger | -0.01 (-0.02, 0.00) | 0.99 (0.98, 1.00) | 2.22E-01 | 4.18E-01 |
|  |  | 395 |  |  | MVMR- weighted median | -0.01 (-0.02, -0.01) | 0.99 (0.98, 0.99) | 4.93E-05 |  |
|  |  | 395 | 77.85 | 1.82E-17 | MVMR-IVW^b^ | -0.01 (-0.02, -0.01) | 0.99 (0.98, 0.99) | 4.62E-06 |  |
| Omega 6 |  | 46 | 4.84 | 5.08E-19 | MVMR-IVW^a^ | -0.07 (-0.20, 0.06) | 0.93 (0.82, 1.06) | 2.79E-01 |  |
|  |  | 46 |  |  | MVMR-Egger | -0.06 (-0.19, 0.06) | 0.94 (0.83, 1.07) | 3.32E-01 | 4.38E-01 |
|  |  | 46 |  |  | MVMR- weighted median | -0.07 (-0.18, 0.05) | 0.94 (0.84, 1.05) | 2.45E-01 |  |
|  |  | 46 | 4.84 | 5.08E-19 | MVMR-IVW^b^ | -0.07 (-0.20, 0.06) | 0.93 (0.82, 1.06) | 2.79E-01 |  |
| Isoleucine |  | 7 | 104.58 | 7.20E-02 | MVMR-IVW^a^ | -0.10 (-0.36, 0.16) | 0.91 (0.70, 1.18) | 4.55E-01 |  |
|  |  | 7 |  |  | MVMR-Egger | 0.56 (0.05, 1.07) | 1.75 (1.05, 2.90) | 3.13E-02 | 5.85E-03 |
|  |  | 7 |  |  | MVMR- weighted median | -0.17 (-0.49, 0.16) | 0.85 (0.61, 1.17) | 3.19E-01 |  |
|  |  | 7 | 104.58 | 7.20E-02 | MVMR-IVW^b^ | -0.10 (-0.36, 0.16) | 0.91 (0.70, 1.18) | 4.55E-01 |  |
| Leucine |  | 12 | 106.74 | 5.33E-03 | MVMR-IVW^a^ | -0.06 (-0.28, 0.16) | 0.94 (0.75, 1.17) | 5.85E-01 |  |
|  |  | 12 |  |  | MVMR-Egger | 0.23 (-0.25, 0.70) | 1.26 (0.78, 2.02) | 3.45E-01 | 1.79E-01 |
|  |  | 12 |  |  | MVMR- weighted median | -0.07 (-0.29, 0.16) | 0.94 (0.75, 1.17) | 5.60E-01 |  |
|  |  | 12 | 106.74 | 5.33E-03 | MVMR-IVW^b^ | -0.06 (-0.28, 0.16) | 0.94 (0.75, 1.17) | 5.85E-01 |  |
| Valine |  | 12 | 148.51 | 1.94E-01 | MVMR-IVW^a^ | -0.07 (-0.20, 0.06) | 0.93 (0.82, 1.06) | 2.96E-01 |  |
|  |  | 12 |  |  | MVMR-Egger | 0.10 (-0.12, 0.32) | 1.11 (0.89, 1.38) | 3.66E-01 | 6.97E-02 |
|  |  | 12 |  |  | MVMR- weighted median | 0.00 (-0.19, 0.20) | 1.00 (0.83, 1.22) | 9.70E-01 |  |
|  |  | 12 | 148.51 | 1.94E-01 | MVMR-IVW^b^ | -0.07 (-0.20, 0.06) | 0.93 (0.82, 1.06) | 2.96E-01 |  |
| Phenylalanine |  | 5 | 2.58 | 3.63E-01 | MVMR-IVW^a^ | 0.09 (-0.09, 0.27) | 1.10 (0.92, 1.32) | 3.08E-01 |  |
|  |  | 5 |  |  | MVMR-Egger | 0.02 (-0.19, 0.23) | 1.02 (0.83, 1.25) | 8.53E-01 | 2.28E-01 |
|  |  | 5 |  |  | MVMR- weighted median | 0.10 (-0.16, 0.36) | 1.11 (0.85, 1.44) | 4.46E-01 |  |
|  |  | 5 | 2.58 | 3.63E-01 | MVMR-IVW^b^ | 0.09 (-0.09, 0.27) | 1.10 (0.92, 1.32) | 3.08E-01 |  |
| Tyrosine |  | 18 | 146.49 | 8.65E-11 | MVMR-IVW^a^ | -0.09 (-0.30, 0.11) | 0.91 (0.74, 1.12) | 3.71E-01 |  |
|  |  | 18 |  |  | MVMR-Egger | 0.16 (-0.12, 0.45) | 1.18 (0.88, 1.56) | 2.64E-01 | 2.27E-02 |
|  |  | 18 |  |  | MVMR- weighted median | -0.23 (-0.43, -0.03) | 0.80 (0.65, 0.97) | 2.45E-02 |  |
|  |  | 18 | 146.49 | 8.65E-11 | MVMR-IVW^b^ | -0.09 (-0.30, 0.11) | 0.91 (0.74, 1.12) | 3.71E-01 |  |
| Alanine |  | 27 | 72.89 | 9.20E-07 | MVMR-IVW^a^ | -0.14 (-0.33, 0.05) | 0.87 (0.72, 1.05) | 1.58E-01 |  |
|  |  | 27 |  |  | MVMR-Egger | 0.05 (-0.51, 0.61) | 1.05 (0.60, 1.84) | 8.63E-01 | 4.87E-01 |
|  |  | 27 |  |  | MVMR- weighted median | -0.07 (-0.26, 0.12) | 0.93 (0.77, 1.12) | 4.63E-01 |  |
|  |  | 26 | 74.40 | 6.82E-05 | MVMR-IVW^b^ | -0.10 (-0.27, 0.08) | 0.91 (0.76, 1.09) | 2.91E-01 |  |
| Glycine |  | 37 | 203.11 | 7.62E-05 | MVMR-IVW^a^ | 0.11 (0.02, 0.20) | 1.12 (1.02, 1.22) | 1.91E-02 |  |
|  |  | 37 |  |  | MVMR-Egger | 0.16 (0.02, 0.29) | 1.17 (1.03, 1.33) | 1.97E-02 | 3.31E-01 |
|  |  | 37 |  |  | MVMR- weighted median | 0.01 (-0.11, 0.13) | 1.01 (0.90, 1.14) | 8.58E-01 |  |
|  |  | 37 | 203.11 | 7.62E-05 | MVMR-IVW^b^ | 0.11 (0.02, 0.20) | 1.12 (1.02, 1.22) | 1.91E-02 |  |
| Body mass index | Atrial  fibrillation | 405 | 74.54 | 3.15E-24 | MVMR-IVW^a^ | 0.30 (0.23, 0.37) | 1.35 (1.26, 1.45) | **2.90E-16** |  |
|  |  | 405 |  |  | MVMR-Egger | 0.27 (0.07, 0.46) | 1.31 (1.07, 1.59) | 7.24E-03 | 7.11E-01 |
|  |  | 405 |  |  | MVMR- weighted median | 0.31 (0.23, 0.39) | 1.36 (1.26, 1.48) | 4.60E-14 |  |
|  |  | 404 | 74.53 | 2.49E-24 | MVMR-IVW^b^ | 0.30 (0.23, 0.37) | 1.35 (1.26, 1.45) | 3.06E-16 |  |
| Height |  | 919 | 256.44 | 5.33E-64 | MVMR-IVW^a^ | 0.29 (0.24, 0.34) | 1.34 (1.27, 1.41) | **1.10E-31** |  |
|  |  | 919 |  |  | MVMR-Egger | 0.33 (0.25, 0.42) | 1.39 (1.28, 1.52) | 1.59E-14 | 2.53E-01 |
|  |  | 919 |  |  | MVMR- weighted median | 0.30 (0.25, 0.36) | 1.36 (1.28, 1.43) | 6.13E-27 |  |
|  |  | 915 | 256.53 | 8.02E-64 | MVMR-IVW^b^ | 0.30 (0.25, 0.35) | 1.35 (1.29, 1.42) | 1.28E-32 |  |
| Appendicular  lean mass |  | 508 | 8.08 | 1.69E-49 | MVMR-IVW^a^ | 0.29 (0.22, 0.36) | 1.33 (1.25, 1.43) | **1.70E-17** |  |
|  |  | 508 |  |  | MVMR-Egger | 0.28 (0.21, 0.35) | 1.33 (1.24, 1.42) | 7.09E-16 | 3.96E-01 |
|  |  | 508 |  |  | MVMR- weighted median | 0.31 (0.24, 0.38) | 1.36 (1.27, 1.47) | 4.00E-17 |  |
|  |  | 499 | 7.22 | 2.14E-47 | MVMR-IVW^b^ | 0.29 (0.22, 0.35) | 1.33 (1.24, 1.42) | 3.76E-17 |  |
| Type 2 diabetes |  | 132 | 86.57 | 2.90E-10 | MVMR-IVW^a^ | -0.01 (-0.04, 0.02) | 0.99 (0.96, 1.02) | 4.53E-01 |  |
|  |  | 132 |  |  | MVMR-Egger | -0.06 (-0.13, 0.01) | 0.94 (0.88, 1.01) | 9.13E-02 | 1.31E-01 |
|  |  | 132 |  |  | MVMR- weighted median | -0.04 (-0.08, -0.01) | 0.96 (0.92, 0.99) | 1.94E-02 |  |
|  |  | 130 | 84.52 | 1.54E-09 | MVMR-IVW^b^ | -0.01 (-0.04, 0.02) | 0.99 (0.96, 1.02) | 6.51E-01 |  |
| Two hours glucose |  | 6 | 58.85 | 9.54E-02 | MVMR-IVW^a^ | -0.16 (-0.31, -0.01) | 0.85 (0.74, 0.99) | 3.80E-02 |  |
|  |  | 6 |  |  | MVMR-Egger | -0.34 (-0.95, 0.26) | 0.71 (0.39, 1.30) | 2.67E-01 | 5.33E-01 |
|  |  | 6 |  |  | MVMR- weighted median | -0.22 (-0.38, -0.06) | 0.80 (0.69, 0.94) | 6.24E-03 |  |
|  |  | 5 | 53.04 | 7.07E-02 | MVMR-IVW^b^ | -0.14 (-0.30, 0.02) | 0.87 (0.74, 1.02) | 8.02E-02 |  |
| Total cholesterol |  | 255 | 299.33 | 1.10E-24 | MVMR-IVW^a^ | -0.01 (-0.07, 0.05) | 0.99 (0.93, 1.05) | 7.59E-01 |  |
|  |  | 255 |  |  | MVMR-Egger | 0.06 (-0.03, 0.16) | 1.07 (0.97, 1.17) | 1.95E-01 | 5.63E-02 |
|  |  | 255 |  |  | MVMR- weighted median | -0.03 (-0.09, 0.04) | 0.97 (0.91, 1.04) | 4.67E-01 |  |
|  |  | 253 | 299.39 | 1.83E-23 | MVMR-IVW^b^ | -0.00 (-0.06, 0.06) | 1.00 (0.94, 1.06) | 9.09E-01 |  |
| Triglycerides |  | 227 | 251.92 | 1.06E-29 | MVMR-IVW^a^ | -0.05 (-0.12, 0.03) | 0.95 (0.89, 1.03) | 2.11E-01 |  |
|  |  | 227 |  |  | MVMR-Egger | -0.09 (-0.20, 0.03) | 0.92 (0.82, 1.03) | 1.45E-01 | 3.90E-01 |
|  |  | 227 |  |  | MVMR- weighted median | -0.04 (-0.12, 0.03) | 0.96 (0.89, 1.03) | 2.67E-01 |  |
|  |  | 226 | 251.49 | 8.10E-29 | MVMR-IVW^b^ | -0.04 (-0.12, 0.03) | 0.96 (0.89, 1.03) | 2.52E-01 |  |
| Systolic  blood pressure |  | 366 | 75.59 | 6.57E-54 | MVMR-IVW^a^ | 0.02 (0.01, 0.02) | 1.02 (1.01, 1.02) | **8.85E-13** |  |
|  |  | 366 |  |  | MVMR-Egger | 0.02 (0.01, 0.03) | 1.02 (1.01, 1.04) | 7.42E-04 | 6.44E-01 |
|  |  | 366 |  |  | MVMR- weighted median | 0.02 (0.01, 0.03) | 1.02 (1.01, 1.03) | 1.89E-12 |  |
|  |  | 366 | 75.59 | 6.57E-54 | MVMR-IVW^b^ | 0.02 (0.01, 0.02) | 1.02 (1.01, 1.02) | 8.85E-13 |  |
| Omega 6 |  | 44 | 4.54 | 9.95E-05 | MVMR-IVW^a^ | -0.06 (-0.13, 0.02) | 0.94 (0.88, 1.02) | 1.18E-01 |  |
|  |  | 44 |  |  | MVMR-Egger | -0.05 (-0.13, 0.02) | 0.95 (0.88, 1.02) | 1.76E-01 | 6.44E-01 |
|  |  | 44 |  |  | MVMR- weighted median | -0.02 (-0.11, 0.07) | 0.98 (0.90, 1.07) | 6.40E-01 |  |
|  |  | 44 | 4.54 | 9.95E-05 | MVMR-IVW^b^ | -0.06 (-0.13, 0.02) | 0.94 (0.88, 1.02) | 1.18E-01 |  |
| Isoleucine |  | 7 | 104.58 | 8.90E-02 | MVMR-IVW^a^ | -0.02 (-0.23, 0.19) | 0.98 (0.79, 1.20) | 8.27E-01 |  |
|  |  | 7 |  |  | MVMR-Egger | 0.11 (-0.46, 0.69) | 1.12 (0.63, 2.00) | 6.98E-01 | 6.12E-01 |
|  |  | 7 |  |  | MVMR- weighted median | -0.11 (-0.38, 0.16) | 0.89 (0.68, 1.17) | 4.12E-01 |  |
|  |  | 7 | 104.58 | 8.90E-02 | MVMR-IVW^b^ | -0.02 (-0.23, 0.19) | 0.98 (0.79, 1.20) | 8.27E-01 |  |
| Leucine |  | 10 | 101.90 | 4.38E-01 | MVMR-IVW^a^ | -0.01 (-0.13, 0.12) | 0.99 (0.87, 1.12) | 8.94E-01 |  |
|  |  | 10 |  |  | MVMR-Egger | -0.01 (-0.29, 0.26) | 0.99 (0.75, 1.29) | 9.16E-01 | 9.60E-01 |
|  |  | 10 |  |  | MVMR- weighted median | -0.01 (-0.18, 0.16) | 0.99 (0.84, 1.17) | 9.09E-01 |  |
|  |  | 10 | 101.90 | 4.38E-01 | MVMR-IVW^b^ | -0.01 (-0.13, 0.12) | 0.99 (0.87, 1.12) | 8.94E-01 |  |
| Valine |  | 12 | 139.09 | 3.10E-01 | MVMR-IVW^a^ | 0.09 (-0.03, 0.21) | 1.10 (0.97, 1.24) | 1.25E-01 |  |
|  |  | 12 |  |  | MVMR-Egger | 0.04 (-0.19, 0.28) | 1.05 (0.83, 1.32) | 7.12E-01 | 6.23E-01 |
|  |  | 12 |  |  | MVMR- weighted median | 0.24 (0.07, 0.41) | 1.27 (1.07, 1.51) | 6.39E-03 |  |
|  |  | 12 | 139.09 | 3.10E-01 | MVMR-IVW^b^ | 0.09 (-0.03, 0.21) | 1.10 (0.97, 1.24) | 1.25E-01 |  |
| Phenylalanine |  | 5 | 2.58 | 6.02E-01 | MVMR-IVW^a^ | -0.09 (-0.21, 0.02) | 0.91 (0.81, 1.02) | 1.20E-01 |  |
|  |  | 5 |  |  | MVMR-Egger | -0.09 (-0.24, 0.05) | 0.91 (0.79, 1.05) | 1.97E-01 | 9.47E-01 |
|  |  | 5 |  |  | MVMR- weighted median | -0.07 (-0.25, 0.12) | 0.94 (0.78, 1.13) | 4.83E-01 |  |
|  |  | 5 | 2.58 | 6.02E-01 | MVMR-IVW^b^ | -0.09 (-0.21, 0.02) | 0.91 (0.81, 1.02) | 1.20E-01 |  |
| Tyrosine |  | 13 | 67.92 | 5.16E-01 | MVMR-IVW^a^ | 0.15 (0.03, 0.27) | 1.16 (1.03, 1.31) | 1.58E-02 |  |
|  |  | 13 |  |  | MVMR-Egger | 0.25 (-0.10, 0.59) | 1.28 (0.90, 1.81) | 1.69E-01 | 5.63E-01 |
|  |  | 13 |  |  | MVMR- weighted median | 0.16 (-0.03, 0.34) | 1.17 (0.97, 1.41) | 1.00E-01 |  |
|  |  | 13 | 67.92 | 5.16E-01 | MVMR-IVW^b^ | 0.15 (0.03, 0.27) | 1.16 (1.03, 1.31) | 1.58E-02 |  |
| Alanine |  | 23 | 68.47 | 6.42E-03 | MVMR-IVW^a^ | 0.05 (-0.08, 0.18) | 1.05 (0.92, 1.20) | 4.33E-01 |  |
|  |  | 23 |  |  | MVMR-Egger | 0.18 (-0.22, 0.57) | 1.20 (0.80, 1.78) | 3.77E-01 | 5.07E-01 |
|  |  | 23 |  |  | MVMR- weighted median | -0.01 (-0.16, 0.14) | 0.99 (0.85, 1.15) | 8.95E-01 |  |
|  |  | 22 | 70.06 | 4.28E-03 | MVMR-IVW^b^ | 0.05 (-0.08, 0.19) | 1.05 (0.92, 1.21) | 4.46E-01 |  |
| Glycine |  | 23 | 153.09 | 1.06E-01 | MVMR-IVW^a^ | -0.01 (-0.09, 0.07) | 0.99 (0.92, 1.07) | 8.29E-01 |  |
|  |  | 23 |  |  | MVMR-Egger | -0.01 (-0.14, 0.11) | 0.99 (0.87, 1.12) | 8.23E-01 | 9.07E-01 |
|  |  | 23 |  |  | MVMR- weighted median | -0.01 (-0.12, 0.10) | 0.99 (0.89, 1.10) | 8.69E-01 |  |
|  |  | 23 | 153.09 | 1.06E-01 | MVMR-IVW^b^ | -0.01 (-0.09, 0.07) | 0.99 (0.92, 1.07) | 8.29E-01 |  |

^a^The inverse variance weighted method was performed without excluding overlapping SNPs between BW and mediators in MVMR analysis.
^b^The inverse variance weighted method was performed after excluding overlapping SNPs between BW and mediators in MVMR analysis.

**Abbreviations:** MVMR, multivariable mendelian randomization


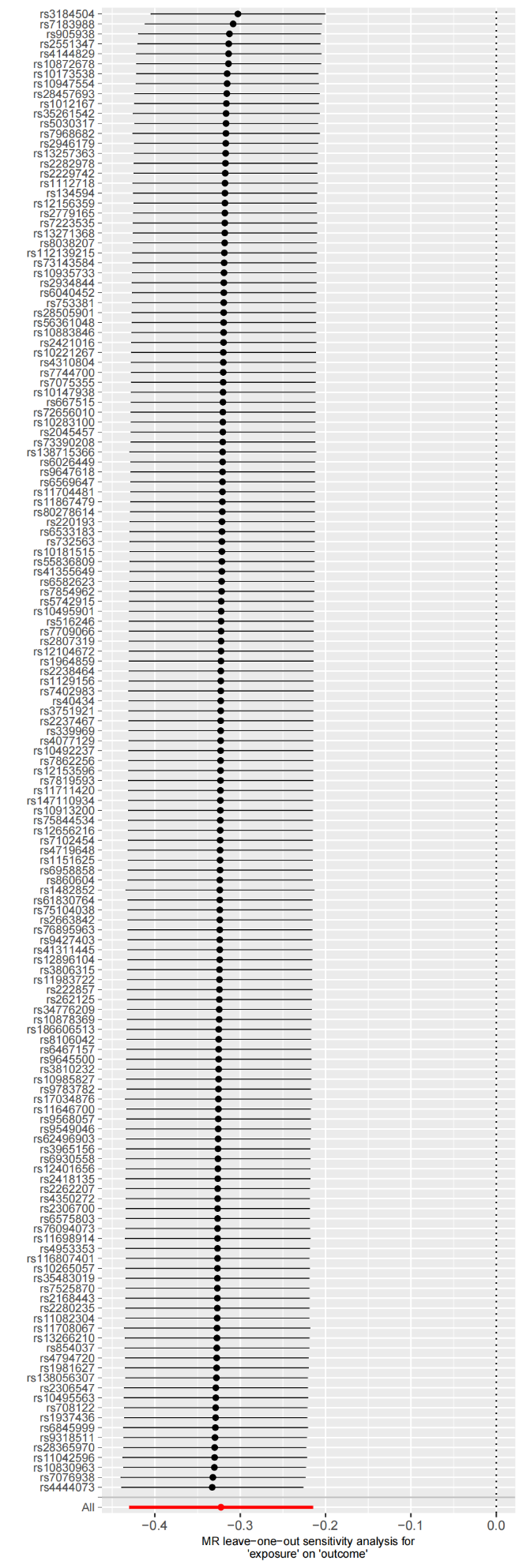


# Supplementary Figure 1. Leave-one-out analysis for the associations between birth weight and coronary heart disease


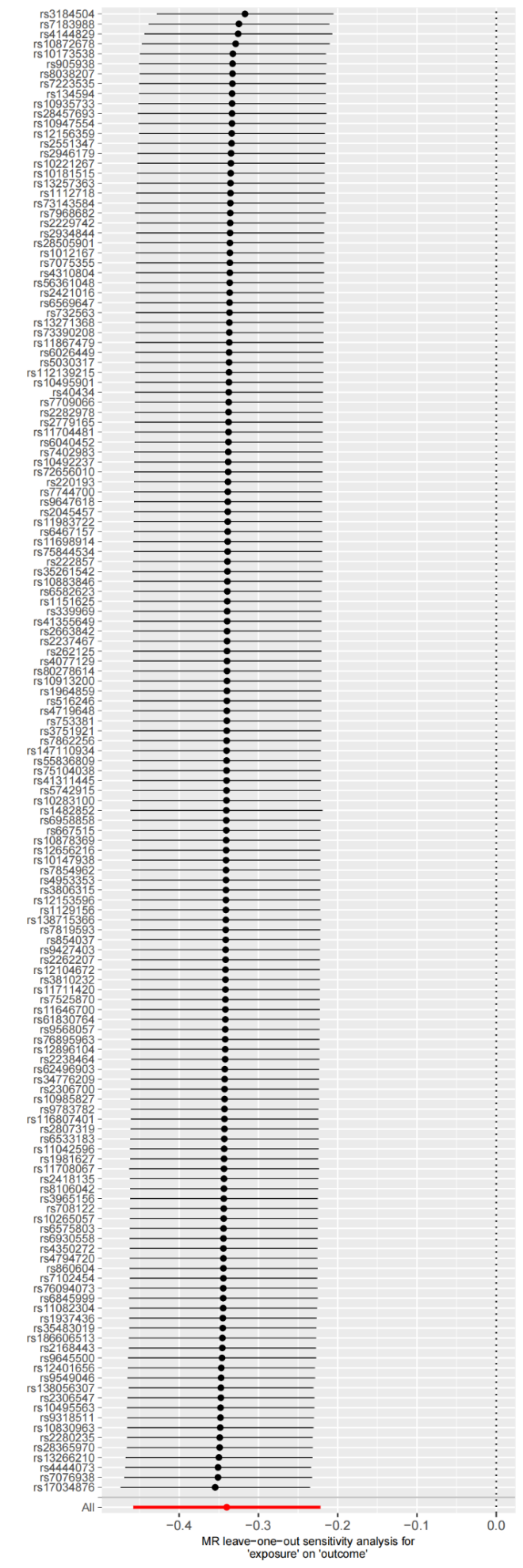


# Supplementary Figure 2. Leave-one-out analysis for the associations between birth weight and myocardial infarction


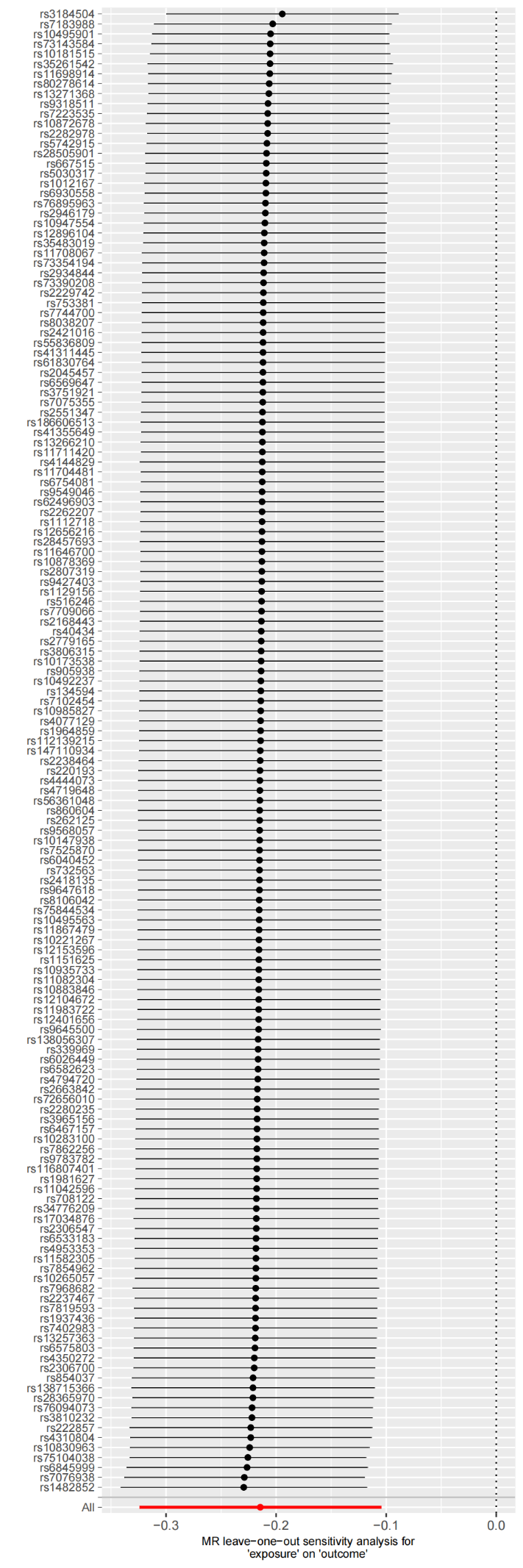


# Supplementary Figure 3. Leave-one-out analysis for the associations between birth weight and angina pectoris


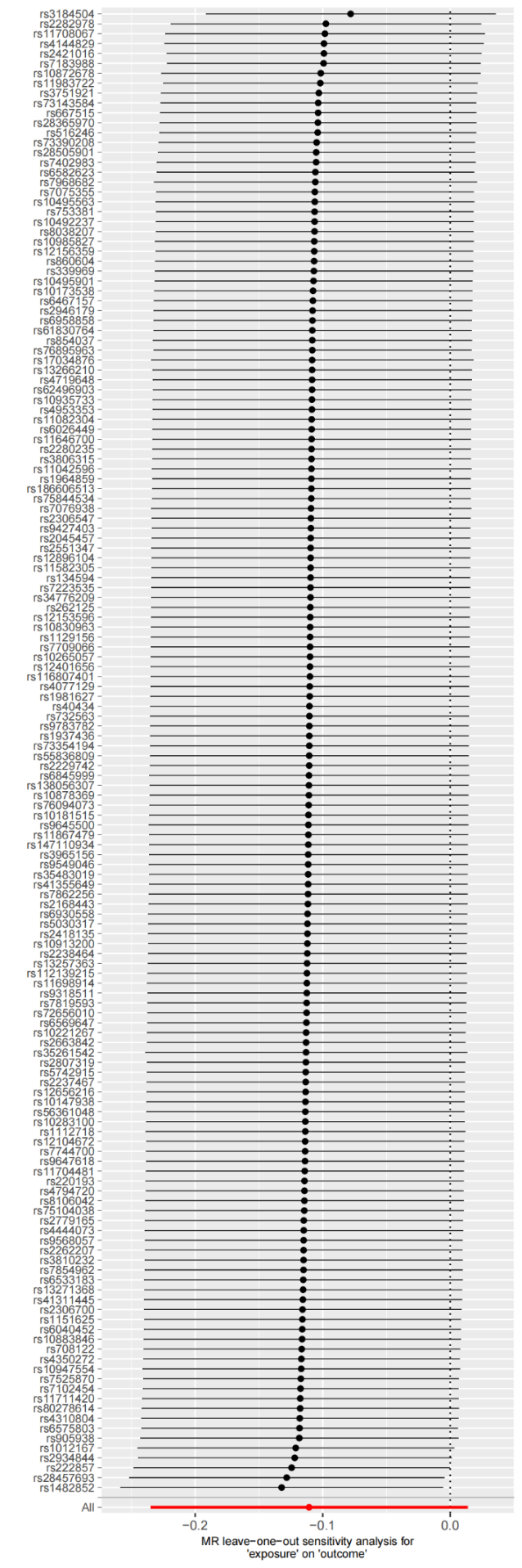


# Supplementary Figure 4. Leave-one-out analysis for the associations between birth weight and ischaemic stroke


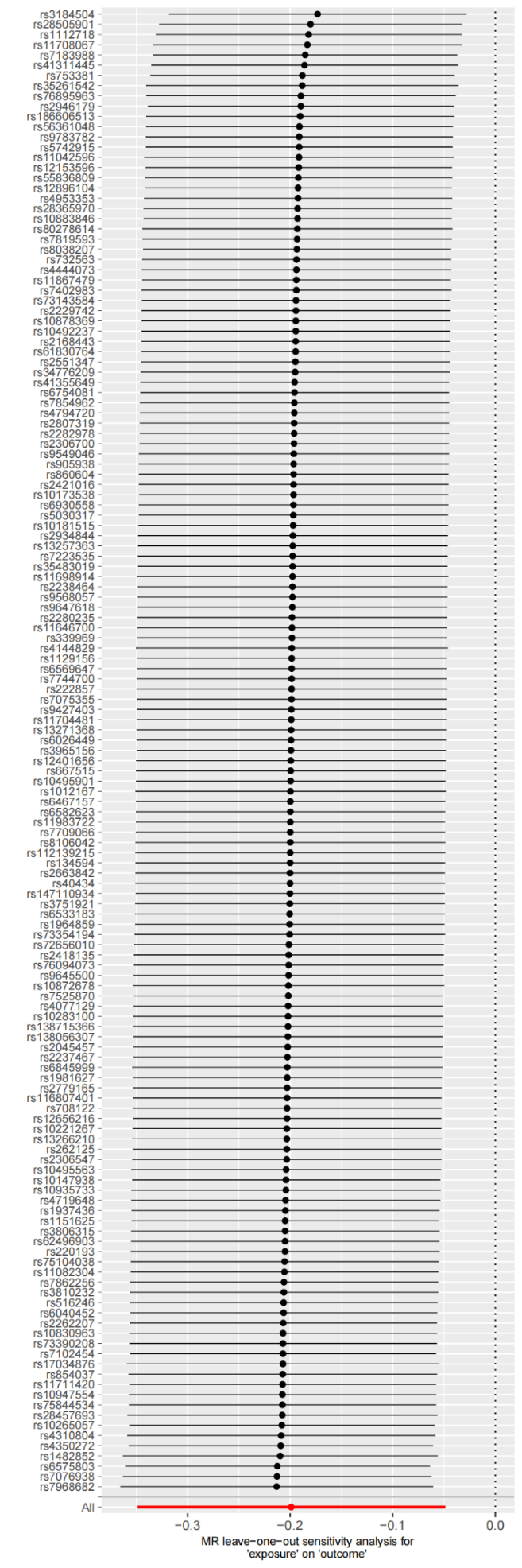


# Supplementary Figure 5. Leave-one-out analysis for the associations between birth weight and peripheral artery disease


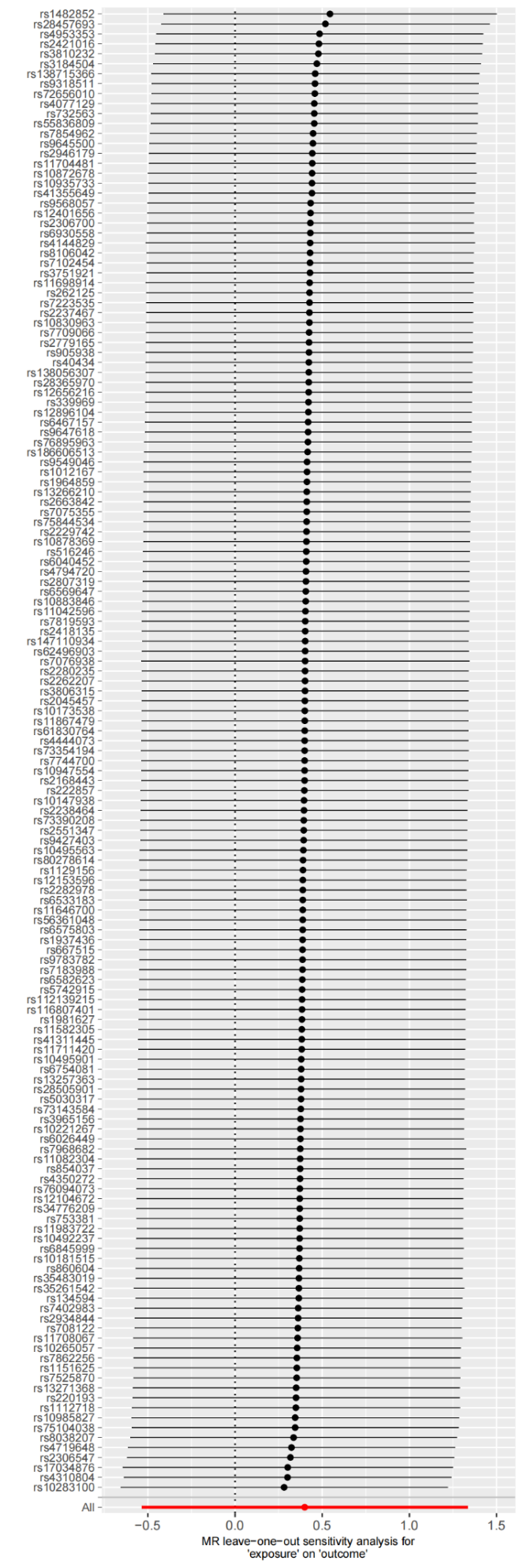


# Supplementary Figure 6. Leave-one-out analysis for the associations between birth weight and rheumatic heart disease


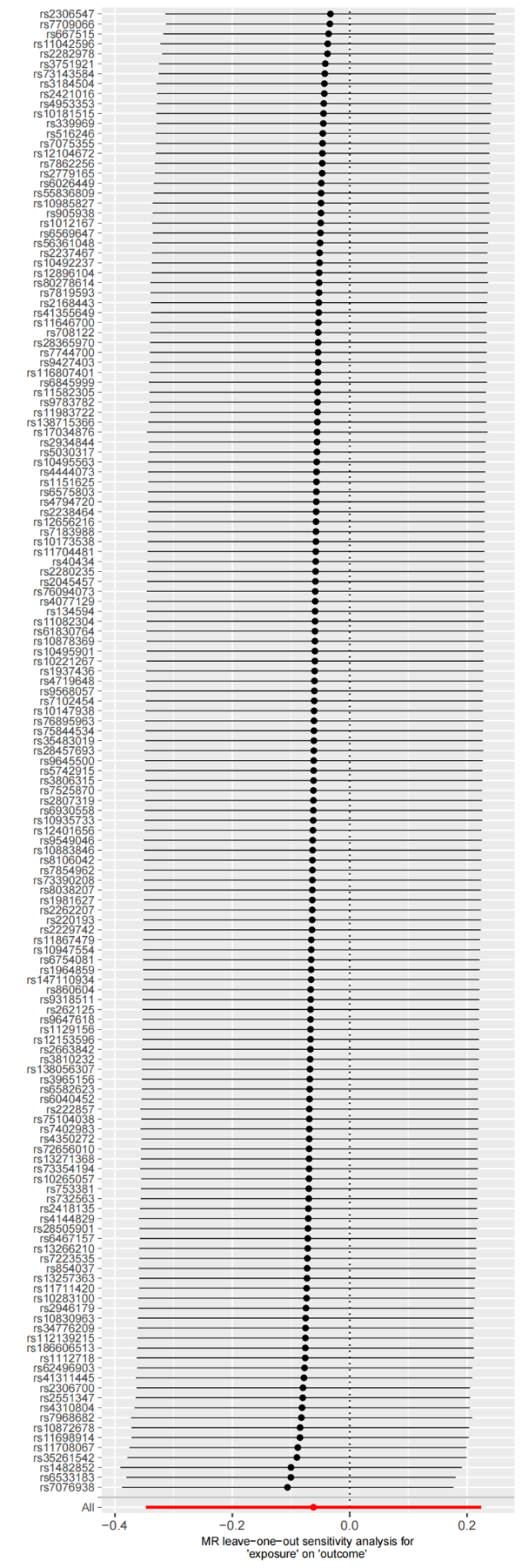


# Supplementary Figure 7. Leave-one-out analysis for the associations between birth weight and myocarditis


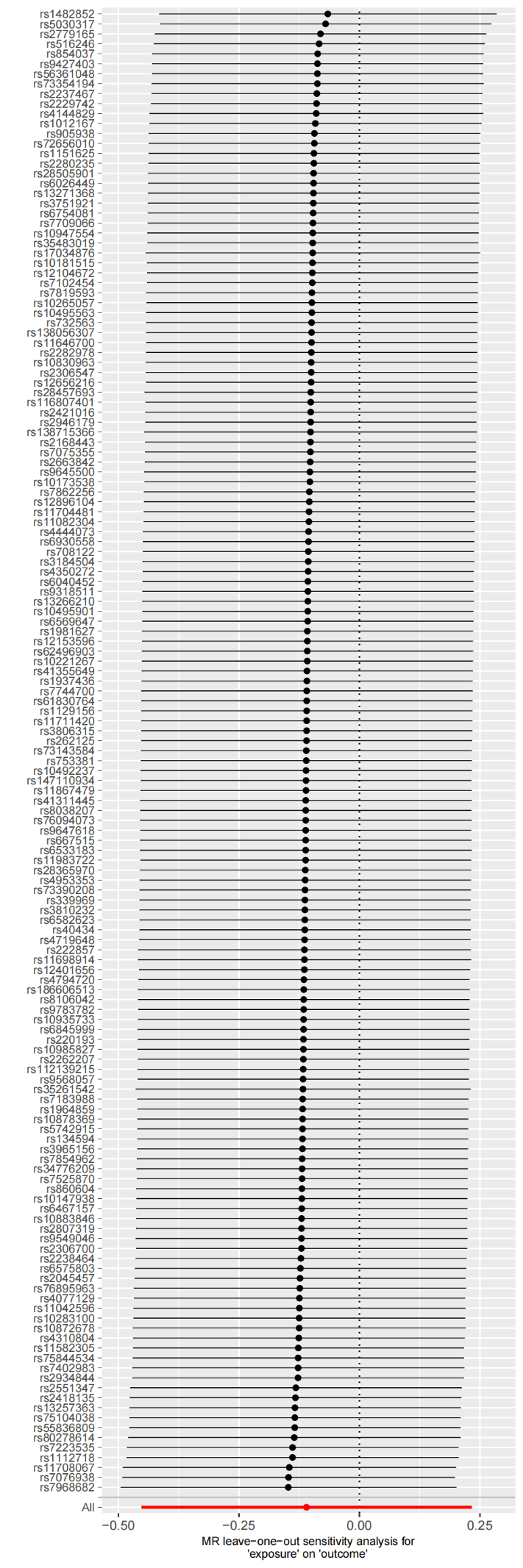


# Supplementary Figure 8. Leave-one-out analysis for the associations between birth weight and pericarditis


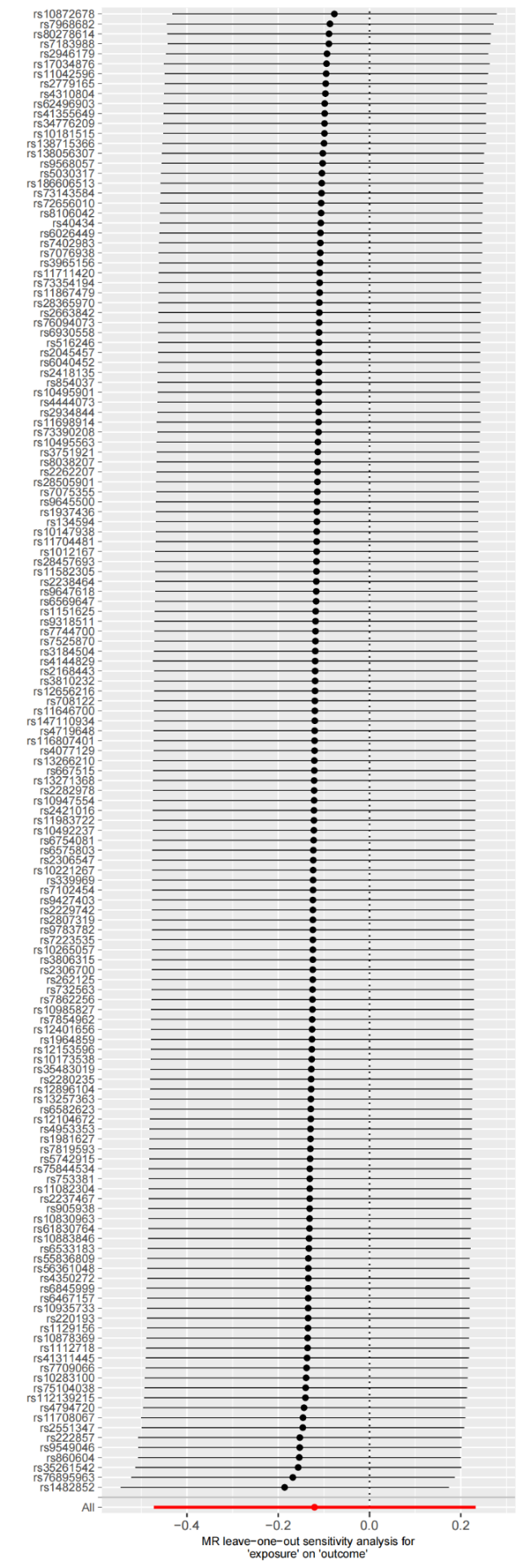


# Supplementary Figure 9. Leave-one-out analysis for the associations between birth weight and endocarditis


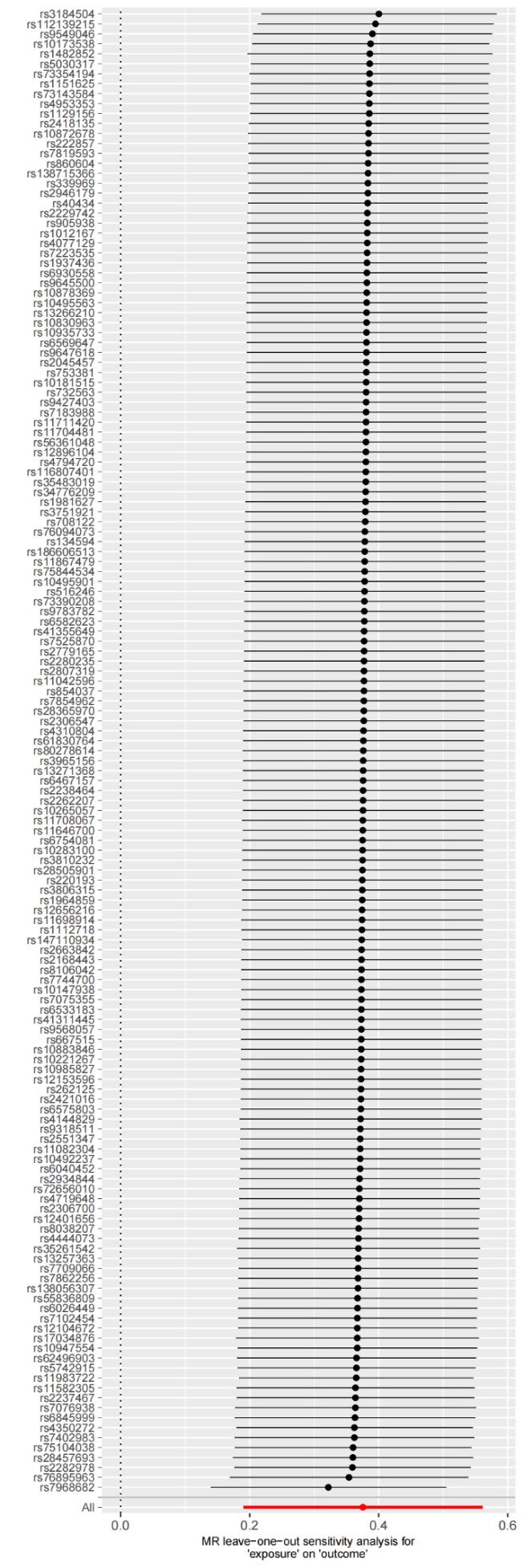


# Supplementary Figure 10. Leave-one-out analysis for the associations between birth weight and aortic aneurysm


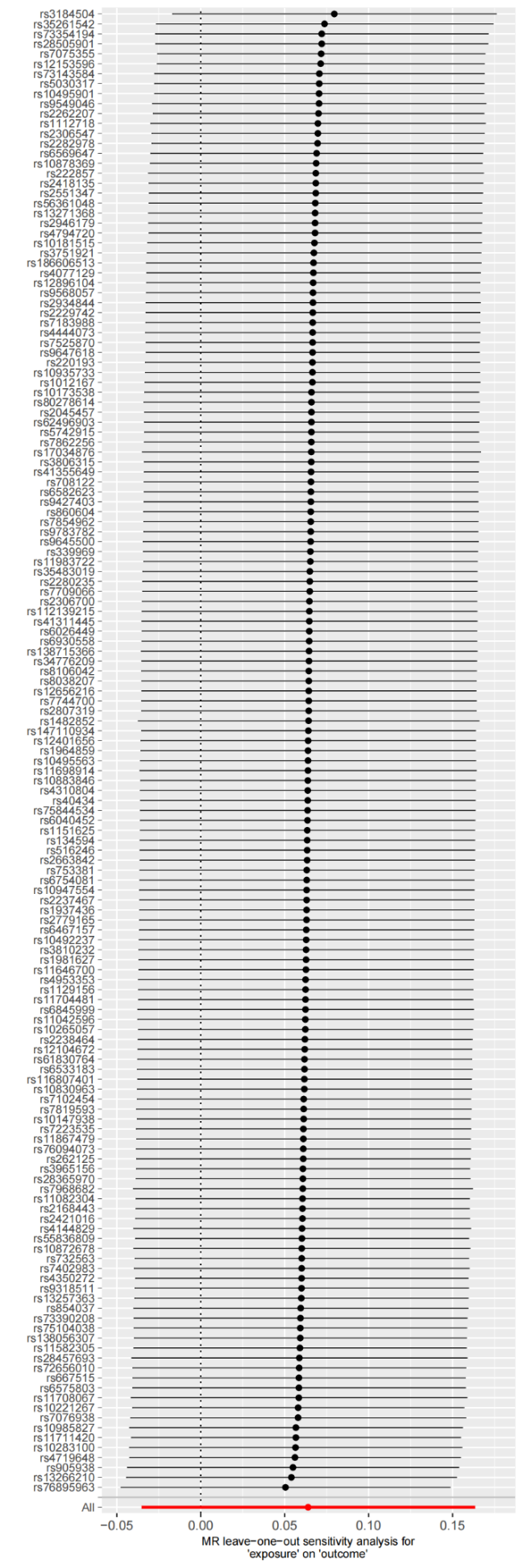


# Supplementary Figure 11. Leave-one-out analysis for the associations between birth weight and heart failure


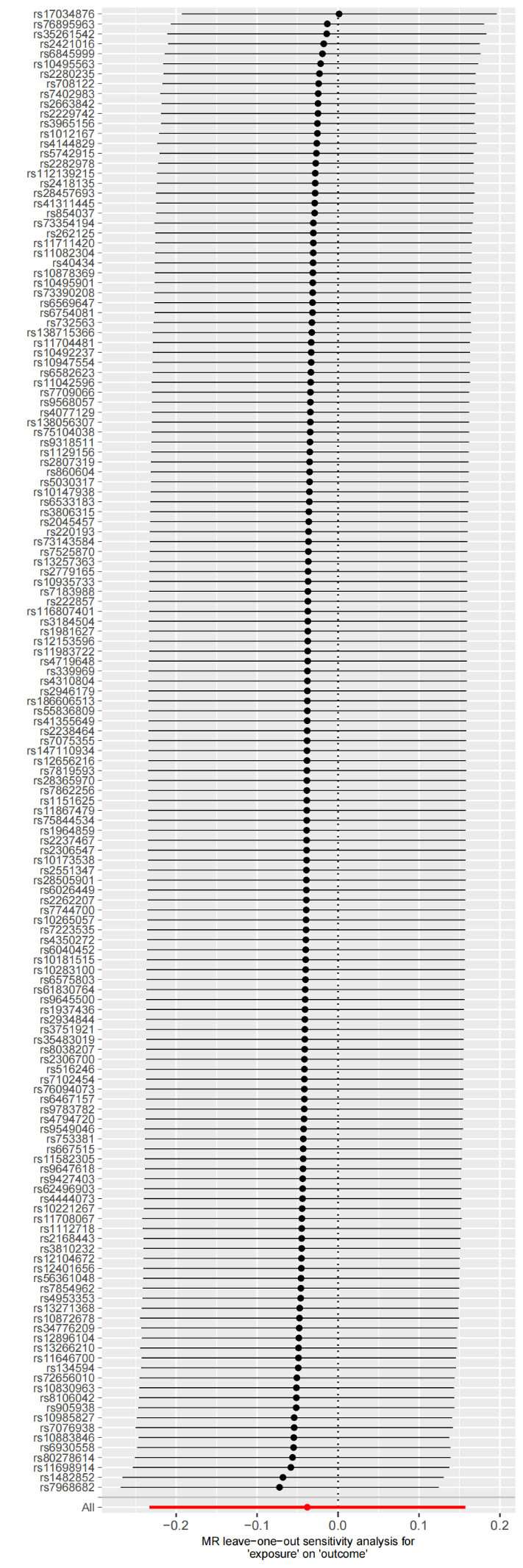


# Supplementary Figure 12. Leave-one-out analysis for the associations between birth weight and intracerebral haemorrhage


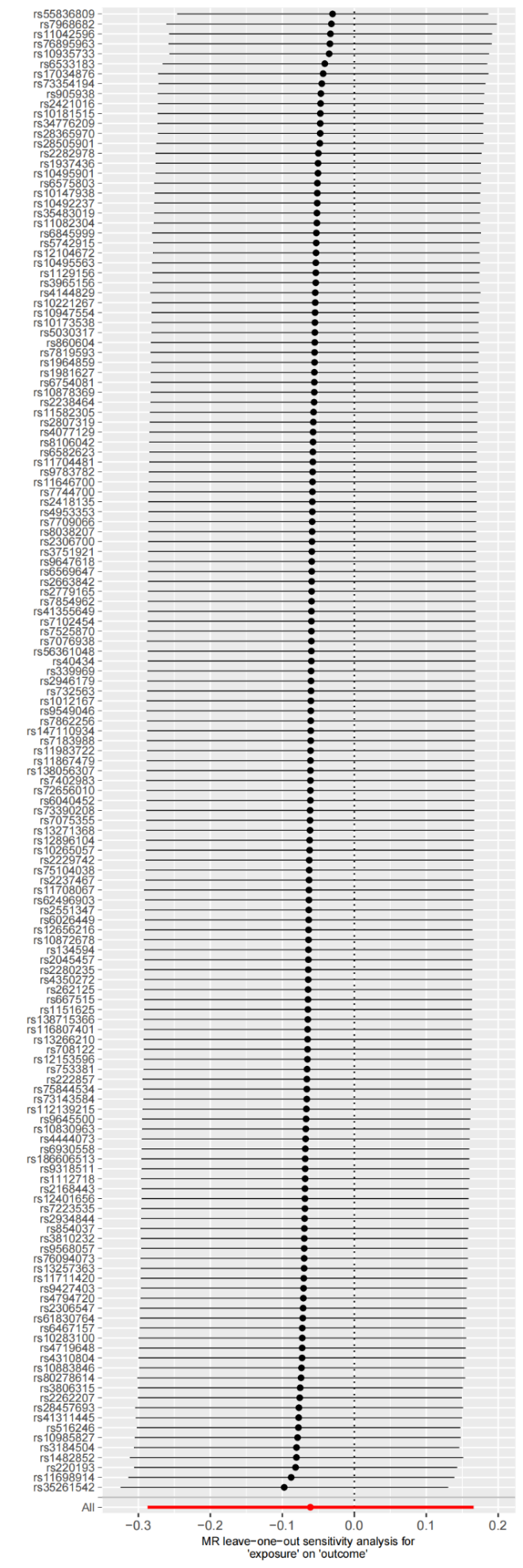


# Supplementary Figure 13. Leave-one-out analysis for the associations between birth weight and subarachnoid haemorrhage


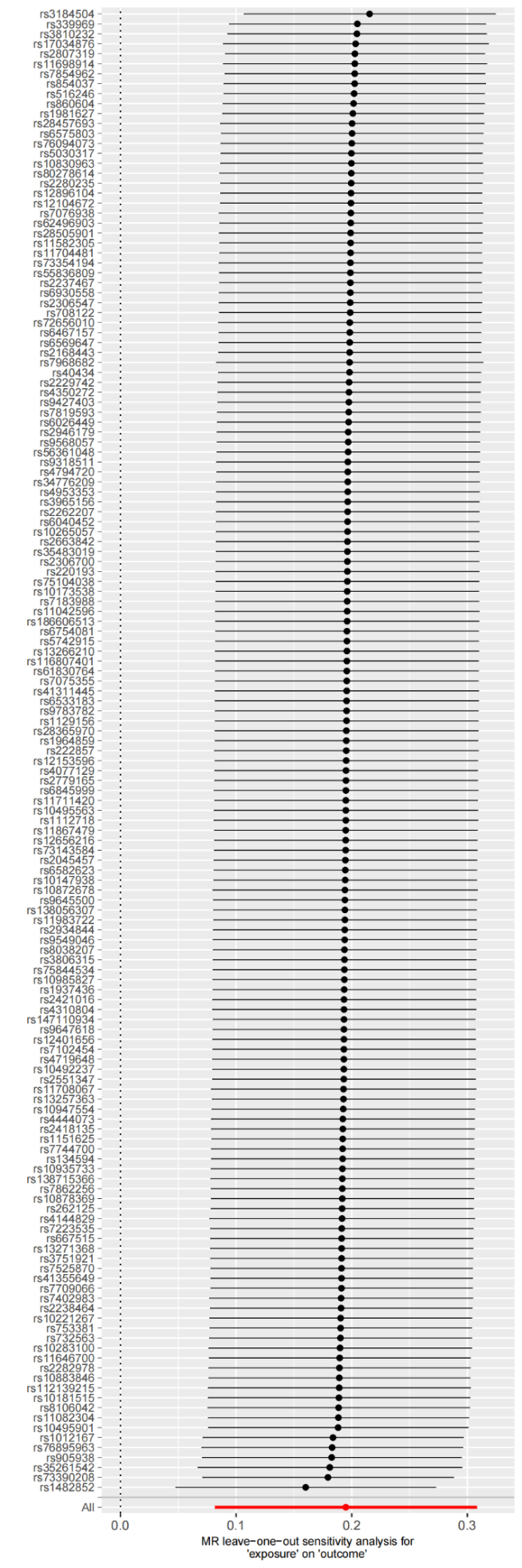


# Supplementary Figure 14. Leave-one-out analysis for the associations between birth weight and venous thromboembolism


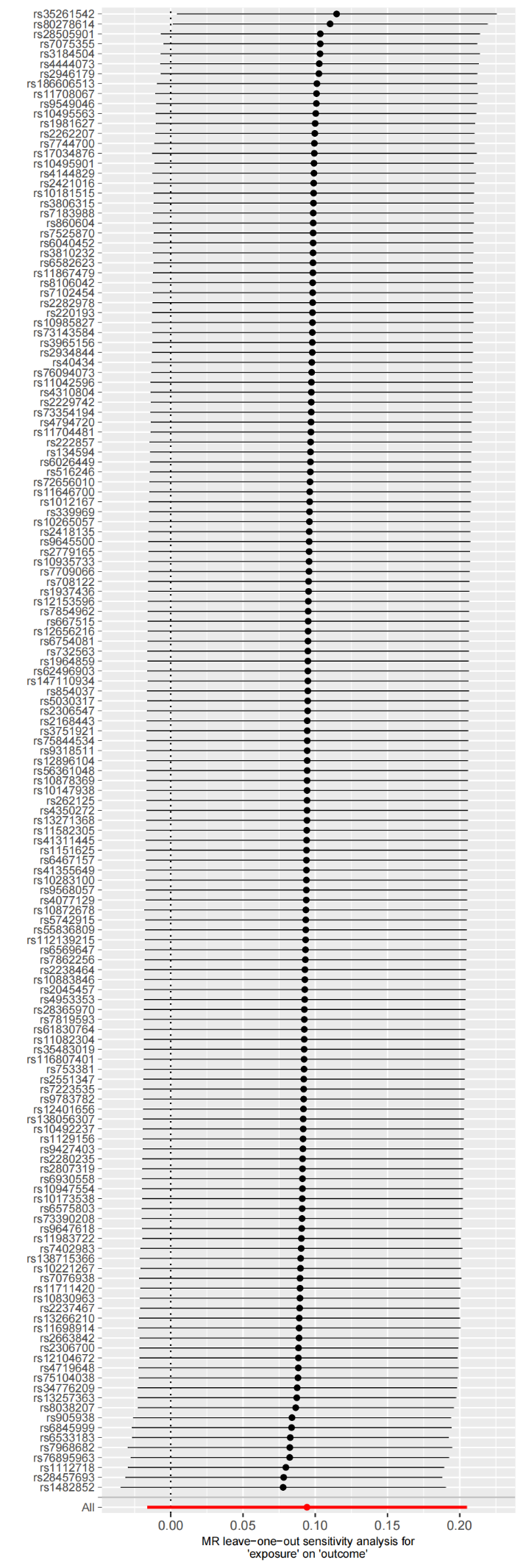


# Supplementary Figure 15. Leave-one-out analysis for the associations between birth weight and non-rheumatic valvular disease


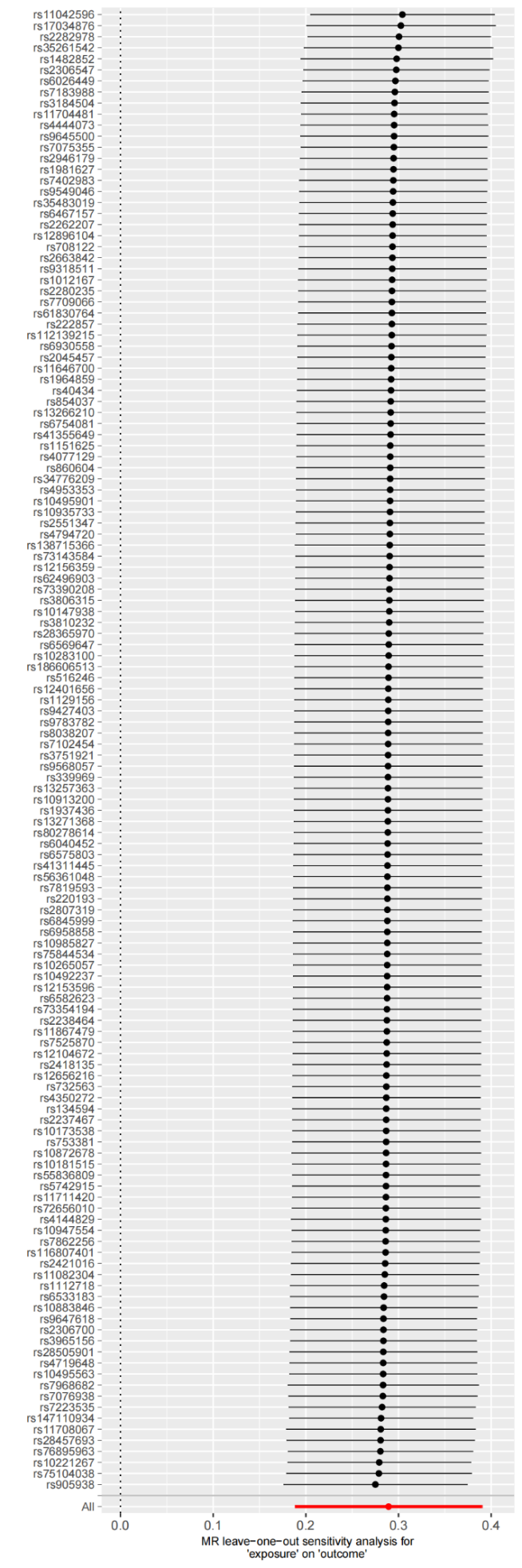


# Supplementary Figure 16. Leave-one-out analysis for the associations between birth weight and atrial fibrillation
