# Supplementary figures and images for: Impact of birth weight on cardiovascular disease and mediating role of metabolic traits: a Mendelian randomisation study
Source: Open Heart. 2025 Oct 31;12(2):e003561. doi: 10.1136/openhrt-2025-003561 (PMC12581085; doi:10.1136/openhrt-2025-003561)

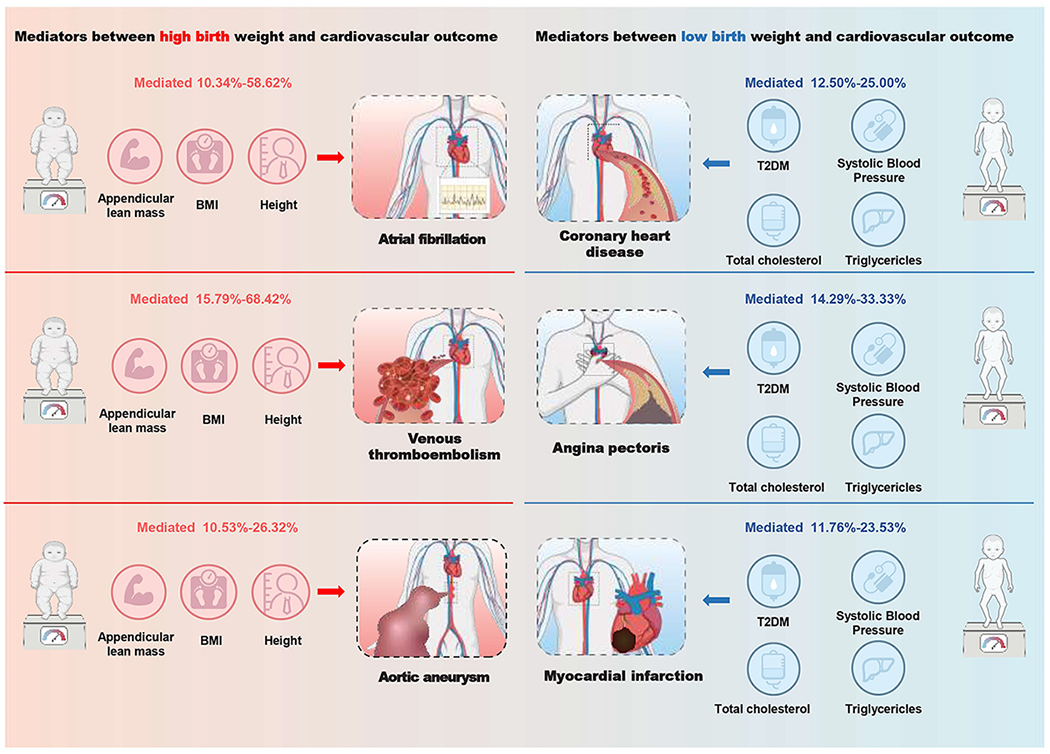

Supplement: online supplemental figure 1 [file openhrt-12-2-s002.tif]
